# The role of juvenile hormone in dominance behavior, reproduction and cuticular pheromone signaling in the caste-flexible epiponine wasp, *Synoeca surinama*

**DOI:** 10.1186/s12983-014-0078-5

**Published:** 2014-10-24

**Authors:** Hans C Kelstrup, Klaus Hartfelder, Fabio S Nascimento, Lynn M Riddiford

**Affiliations:** Janelia Research Campus, Howard Hughes Medical Institute, Ashburn, VA 20147 USA; Faculdade de Medicina de Ribeirão Preto, Universidade de São Paulo, Universidade de São Paul, Av. Bandeirantes 3900, Ribeirão Preto, 14049-900 SP Brazil; Departamento de Biologia da Faculdade de Filosofia, Ciȇncias e Letras de Ribeirão Preto, Universidade de São Paulo, Av. Bandeirantes 3900, Ribeirão Preto, 14040-900 SP Brazil; Present address: Department of Botany and Zoology, Stellenbosch University, Private Bag XI, Matieland, 7602 South Africa

**Keywords:** Cuticular hydrocarbons, Ecdysteroids, Endocrine, Epiponini, Juvenile hormone, Swarm founding, Wasps

## Abstract

**Background:**

The popular view on insect sociality is that of a harmonious division of labor among two morphologically distinct and functionally non-overlapping castes. But this is a highly derived state and not a prerequisite for a functional society. Rather, caste-flexibility is a central feature in many eusocial wasps, where adult females have the potential to become queens or workers, depending on the social environment. In non-swarming paper wasps (e.g., *Polistes*), prospective queens fight one another to assert their dominance, with losers becoming workers if they remain on the nest. This aggression is fueled by juvenile hormone (JH) and ecdysteroids, major factors involved in caste differentiation in most eusocial insects. We tested whether these hormones have conserved aggression-promoting functions in *Synoeca surinama*, a caste-flexible swarm-founding wasp (Epiponini) where reproductive competition is high and aggressive displays are common.

**Results:**

We observed the behavioral interactions of *S. surinama* females in field nests before and after we had removed the egg-laying queen(s). We measured the ovarian reproductive status, hemolymph JH and ecdysteroid titers, ovarian ecdysteroid content, and analyzed the cuticular hydrocarbon (CHC) composition of females engaged in competitive interactions in both queenright and queenless contexts. These data, in combination with hormone manipulation experiments, revealed that neither JH nor ecdysteroids are necessary for the expression of dominance behaviors in *S. surinama*. Instead, we show that JH likely functions as a gonadotropin and directly modifies the cuticular hydrocarbon blend of young workers to match that of a reproductive. Hemolymph ecdysteroids, in contrast, are not different between queens and workers despite great differences in ovarian ecdysteroid content.

**Conclusions:**

The endocrine profile of *S. surinama* shows surprising differences from those of other caste-flexible wasps, although a rise in JH titers in replacement queens is a common theme. Extensive remodeling of hormone functions is also evident in the highly eusocial bees, which has been attributed to the evolution of morphologically defined castes. Our results show that hormones which regulate caste-plasticity can lose these roles even while caste-plasticity is preserved.

**Electronic supplementary material:**

The online version of this article (doi:10.1186/s12983-014-0078-5) contains supplementary material, which is available to authorized users.

## Background

Division of labor, which is the fundamental condition for the ecological success of social insects, reaches its apex with the evolution of morphologically specialized queens and workers. These distinct phenotypes separate the highly advanced eusocial species from the ‘primitively eusocial’ ones, such as *Polistes* paper wasps, where caste is not determined until the adult stage. Yet caste totipotency is not always lost as a consequence of increased social specialization. For example, many of the Neotropical swarm founding wasps (Epiponini) – which are permanently social and polygynous, i.e. have multiple queens in their nests – have retained extreme caste flexibility [1-3]. As has been shown in *Metapolybia aztecoides* and *Synoeca surinama*, the social context in which a young adult female finds herself is the overwhelming factor in determining her caste fate [1-3]. So long as there are queens present and the colony does not split (i.e. form a reproductive swarm), young females will almost certainly become workers, a fate assured by direct suppression by older workers [2-5]. If a colony becomes queenless, the youngest cohort of females on the nest – which would have otherwise become workers – become the next group of queens [3]. The high frequency of sporadic nest destruction (e.g., by army ants or birds) or abandonment has placed a premium on being able to expeditiously adjust the ratio of egg layers to workers to avoid colony extinction [1-3,5,6]. Thus, caste identity is provisional, since queens may transform into workers when worker numbers are low, and young workers (e.g. builders) can become queens if queens disappear [1-3].

**Figure 1:**
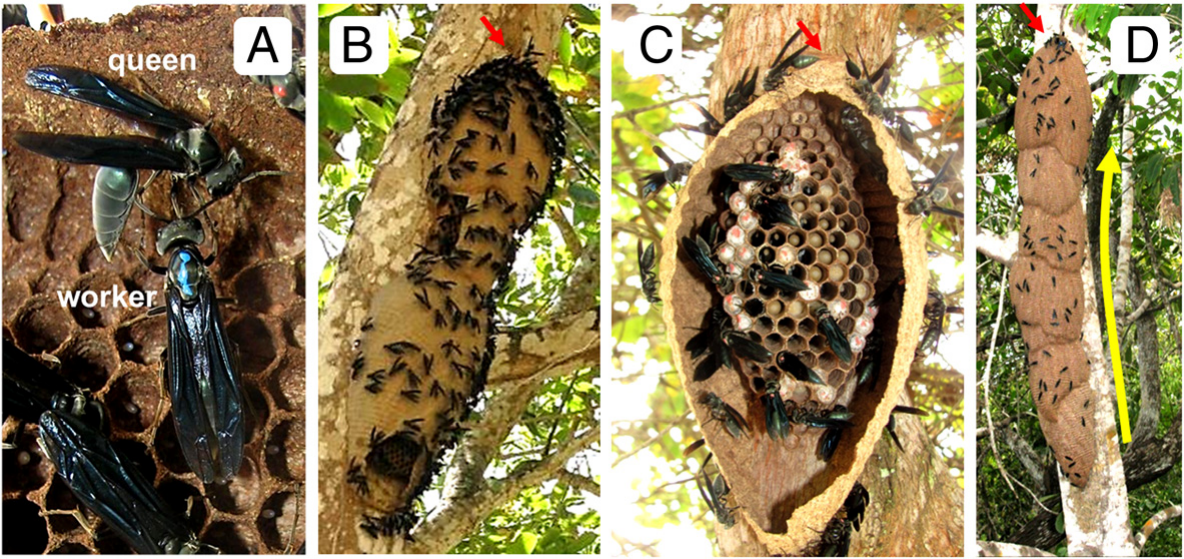
**Nests of** ***Synoeca surinama.*** **(A)** Worker performing the “queen dance” and the queen responding with a pronounced bending display. **(B)** Aggressive defense response in a three compartment nest. The hole in the side was an observation window, and the opening on the bottom was incidental, leading to the emergence of colony defenders. **(C)** A single compartment nest with the envelope removed. Most nests were studied this way. **(D)** A massive 6 or 7 compartment nest discovered in Chapada Diamantina, Bahia, Brazil. Nest expansion proceeds upwards (yellow arrow). Red arrows indicate nest entrance/exit in B-D.

The question of caste determination in wasps has been best studied in temperate climate paper wasps of the genus *Polistes*, but these are not continuously social, as nests are independently founded by overwintered gynes (young queens that do not yet head a colony). These may either found a nest individually or as a group of females. In the latter case, a dominance hierarchy is established primarily through direct acts of aggression and differential oophagy, which typically results in a monogynic society [7-10]. Queens of some caste totipotent epiponines maintain their position through ritualized displays of dominance [11], namely abdomen-bending (or “bending”), a lateral hinging at the waist which bears a resemblance to a stinging posture [2,3] (Figure 1A). Queens display aggressively toward one another, often on the margins of the nest, but bending is also elicited by the approach of workers who engage in a ritualized act of their own: the spasmodic ‘queen-dance’, interpreted as an act of “arrested or inhibited aggression” [1] which, if released, can give way to attacks toward subordinate behaving queens [1-3]. Defeated queens, after passing through a period of idleness [1], may become workers until eventually only one queen remains (which has the effect of increasing relatedness within an otherwise polygynous society) [1,3,12]. Possible selective forces at work (e.g., mutualism, kin selection) in swarm-founding wasp societies have been discussed [1,3,13], but what remains to be investigated are the proximate mechanisms (i.e., behavioral and physiological modifiers) that underlie their extraordinary caste flexibility and performance.

In insects, the most frequently employed factor for orchestrating differentially expressed phenotypes (i.e., polyphenisms) is juvenile hormone (JH) [14], a sesquiterpenoid. In addition to its widely conserved roles in pre-imaginal development [15,16], JH is also a widespread gonadotropin [17,18]. In *Polistes* wasps, caste determination and maintenance is modulated by context- and nutrition-dependent effects of JH [19-22]. Among competing foundresses, JH drives ovarian growth and aggression [23-25], its hemolymph titers remain high in queens [21,26], and it is tightly linked to the production of chemical signatures of fertility in the cuticular hydrocarbon (CHC) profile [27]. Ecdysteroids, which in adult female insects are primarily produced by the ovaries and loaded into growing oocytes, may also be released into the hemolymph, as is the case in *Polistes.* They augment dominance in competing foundresses [24,25,28] but are not required to maintain dominance behaviors [29]. In *Polistes* females emerging in queenright conditions, methoprene (a stable JH mimic) treatments induced precocious worker behaviors, such as guarding or foraging [21,30]. Yet if these otherwise worker-destined females are removed from their environment and fed *ad libitum*, methoprene treatments resulted in enhanced oocyte development [19], and indeed, queenless workers that fight their way up the dominance hierarchy have especially high amounts of JH [26].

*Polistes* and epiponine wasps share a common caste-based ancestor [31] which was also likely to be caste-totipotent in the adult stage [32], setting the expectation for conserved functions for JH and the ecdysteroids in both caste transition and the subsequent modulation of caste physiology and behavior. Yet aside from the evidence that methoprene accelerates the onset of worker behaviors in the epiponine wasp *Polybia occidentalis* [33], the JH and ecdysteroid profiles in another caste-flexible wasp, *Polybia micans,* were remarkably different from that of *Polistes dominula* [34]. In *P. micans*, both JH and ecdysteroid titers were low in competing queens while JH increased only after all direct competitors were eliminated. Neither hormone thus appears to be essential for maintaining basic queen physiology, be it ovarian growth or chemical signaling. Despite these differences, JH titers were increased in potential reproductives following queen removal in both *P. micans* [34] and *P. dominula* [26], suggesting that JH fuels competitive ability and/or increases reproductive potential in challenging, unstable conditions, analogous to testosterone function in vertebrates [35,36].

The observed loss of hormone function with increasing level of sociality is paralleled in other lineages of Hymenoptera [14]. In the adults of primitively eusocial bumble bees, circulating JH and ecdysteroids are important for ovarian growth and reproductive behaviors [37-39]. Yet in swarm-founding, caste-dimorphic honey bees and stingless bees, these hormones have no obvious functions for reproduction or related behaviors [14,40]. Significant modification of JH function in eusocial Hymenoptera does not always accompany the switch to a swarming lifestyle, as JH has also relinquished gonadotropic functions in some ants [14,41]. The reason for convergent loss of hormone functions among these eusocial lineages remains a mystery, but the wasps are the best candidates for tracking the mechanisms of *how* these transitions occurred. Although there are no extant intermediate representatives between bumble bees and swarming-founding bees, there are many genera phylogenetically intermediate to *Polistes* and *Polybia* [31].

*Synoeca surinama*, whose social biology is very similar to the closely related *Metapolybia* [1], is a derived epiponine wasp, as is *Polybia* [31]. As opposed to *P. micans*, where queen selection involves, in part, an onslaught of physical attacks [34], in colonies of *Synoeca* queen succession is usually peaceful: even newly emerged females, which are relatively soft-bodied, can assume bending and gain acceptance as queens without a fight [1]. We show that the endocrinology of *S. surinama* is distinct from *Polistes* and *Polybia*, indicating that hormone functions in social wasps are liable to evolutionary change, even as caste-totipotency is conserved. In addition, the CHC components, including prospective queen signals, have diverged considerably between these two swarm founding wasps, possibly reflecting strong intragroup selection for the Epiponini as compared to some temperate wasps [42].

## Results

### Colony demographics and behavior

In total, 12 colonies were studied (Table 1). Queen number ranged from 1–32 while worker number ranged from approximately 65 to 300. All nests had produced at least one generation of females (i.e., were post-emergent colonies). Colony 6, the single pre-emergent colony studied, had founded their nest at least 4 weeks prior, indicating that all females were at least that old. Colonies 4–10 were first observed when in a queenright condition and, following queen removal, in a queenless state (Table 1).

**Table 1:** **Basic information for colonies used in this report**

**Colony**			**Original queens**			**Prospective queens**		**Workers**	**Collection**
**ID**	**Colony phase**	**Envelope removed?**	**#**	**Ctlr. age** ^**a**^	**Oocytes >70% LME** ^**b**^	**♀ ♀ present** ^**c**^	**♀ ♀ benders** ^**d**^	**#**	**Month/Year**
1	post-em’	No	n.d.	n.d.	n.d.	n.a.	n.a.	~200	Feb. 2010
2	post-em’	No	n.d.	n.d.	n.d.	n.a.	n.a.	~300	Mar. 2010
4	post-em’	Yes	4	5	9 (5–12)	W	W	~65	July 2010
5	post-em’	Yes	32	1.5-4	3.4 (1–4)	P, W	P, W	~300	Nov. 2010
6	pre-em’	Yes	12	4.5-5	7.9 (4–10)	W	W	~200	May 2011
7	post-em’	Yes	7	4.5-5	6.4 (4–9)	P, I, W	P	~90	June 2011
8	post-em’	Yes	11	4.5-5	6.5 (5–8)	P, W	P	~150	Oct. 2011
9	post-em’	Yes	1	5	13	P, I, W	P, I, W	~70	Nov. 2011
10	post-em’	Yes	4	4.5	14.8 (10–18)	P, I, W	P, I, W	~250	Nov. 2011
11	post-em’	Yes	1	n.a.	n.d.	n.a.	n.a.	~100	Nov. 2011
12	post-em’	Yes	5	n.a.	n.d.	n.a.	n.a.	~200	Nov. 2011

Colony phase indicates whether the nest had produced the first generation of adults [(pre- and post-emergent (em’)]. Males were only seen in Colony 4. When all queens were removed from a nest, they could be replaced by: 1) females that were still pupae (P) at that time; 2) the youngest adults on the nest which were not observed to work and were idle (I); and/or 3) workers (W).

^a^Age of the wasps is represented as cuticular (Ctlr.) age which was estimated on a scale of 1–5 according to the darkness of the cuticle. Scale shown in Additional file 9: Figure S1.

^b^Average number of oocytes >70% length of mature egg (LME) is given along with the range within a given colony.

^c^♀ ♀ present indicates the types of females present when the queen was removed.

^d^♀ ♀ benders indicates which females became the next cohort of prospective queens.

*n.a.*, Not applicable; *n.d.*, Not determined.

In queenright nests where the envelope was removed but had not been manipulated otherwise (i.e. queen removal), no female was observed to work until at least two days after eclosion, and all females had begun building (envelope reconstruction) once they were four days old (N = 186 from 4 colonies). None of these females were observed to forage before building. Females that were not queens were never seen to lay an egg, but queens (i.e. egg-laying females that frequently clustered together, walked slowly and engaged in bending displays) were occasionally seen to participate in building activities. For instance, on three occasions fully established mated queens from colony 5 (Table 1) were observed to add a ball of pulp to the nest envelope.

When queens were removed, the next cohort of potential queens (i.e., females that engaged in bending displays) usually included the youngest adults present (idle females and workers) and/or those that had emerged after queen removal (Table 1). The number of females permitted to become queens is regulated in part by physical aggression from worker nestmates [1,3]. For example, those females which emerged subsequent to the cohort of accepted benders were attacked (e.g., were mounted and mandibulated without causing ostensible injury) by older nest mates (2 colonies; see below). Even in queenright colonies with established queens, young idle females were observed to receive aggression when walking across the comb (N = 8 from 3 colonies). Finally, in a nest made monogynic by the removal of three queens (which presumably led to an abrupt drop in queen-derived signals in the colony), all young idle females which initiated bending (0–2 days old; N = 10; colony 10) were chased and attacked by the remaining queen and several workers.

### Ovarian development in young females from queenright and queenless colonies

**Figure 2:**
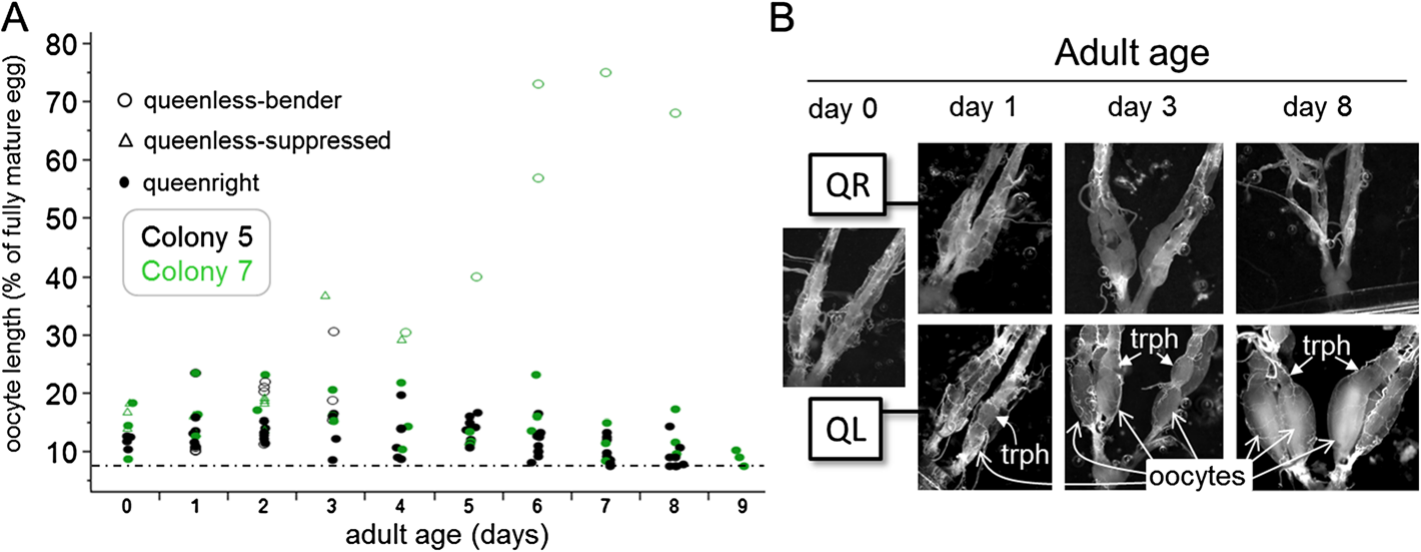
**Ovarian development in young females. (A)** Time course of ovarian maturation in young queenright (QR) and queenless (QL) females from two nests. The average of the longest two primary oocytes is indicated, so that each point represents an individual ovary. Dotted line indicates filamentous ovaries lacking measurable oocytes (<8% LME). **(B)** Typical ovaries of a newly emerged wasp (day 0) and those of older queenright and queenless females. Oocytes and trophocytes (“trph”) are indicated.

Oocyte length (given as % length of mature egg; LME) was assessed in young queenright (worker-destined) and queenless (queen-destined) females from colonies 5 and 7 (Figure 2). In both colonies, the ovaries of queenright workers showed slight maturation over the first few days, before fully regressing as early as day 7 following eclosion (oocytes <8% LME were considered filamentous and not measurable). Oocytes of queenless, unsuppressed benders began to differ from those of queenright females around the adult age of days 2 or 3 (colony 5) (Figure 2A).

### JH titers

#### Correlation with caste

**Figure 3:**
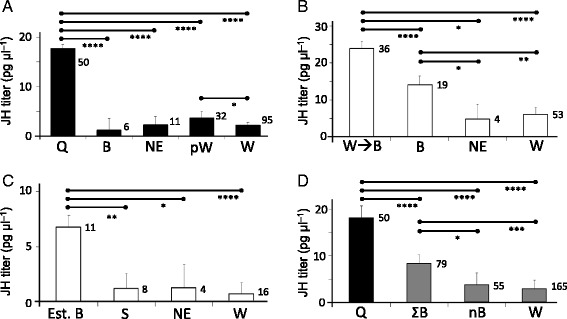
**JH titers according to female status.** A Mixed Model analysis of samples taken from colonies 4–10, using up to two fixed factors (Female Status and Colony Condition) and a random one (Colony). Black, white and gray bars indicate samples from queenright (QR) conditions, queenless (QL) conditions and a combination of samples from QR and QL conditions, respectively. Least Squared Means (±SE) are shown along with sample size. **(A)** In QR conditions, JH differed between the female groups (F4,186=63.29, P<0.0001). Queens (Q) had higher titers than newly emerged (NE) females (t=−8.34), new benders without history of working (B) (t=−6.52), 1–3 day old pre-working and working females (pW) (t=−9.85), and workers >3 days old (W) (t=−15.35)). Also, pW females had higher JH titers than workers (t=−2.14). **(B)** In QL conditions, 1–3 days after queen removal, JH levels differed between the female groups (F3,109=20.32, P<0.0001). Workers that transitioned into benders (W➔B) had higher JH titers than NE females (t=−4.09), bending females which were never observed to work (t=−2.61), and workers (t=−7.53). The latter bending females also had higher JH titer than NE females (t=−2.13) and workers (t=−2.75). **(C)** In QL conditions, 7–8 days after queen removal, JH differed between the female groups (F3,36=5.73, P=0.003). Established benders (Est B) had higher JH titers than all female groups (vs. NE females (t=−2.42); vs. suppressed (S) females (i.e., attacked benders and huddlers) (t=−3.23); vs. workers (t=−3.85). **(D)** When QR and QL groups were pooled, with females classified as either queens, non-queen benders of types (ΣB), 0–3 day old non-benders (nB) and workers, JH differed according to Female Status (F3,341=65.83, P<0.0001), Colony Condition (F1,341=16.18, P<0.0001) and their interaction (F2,341=11.33, P<0.0001). Queens had higher JH titers than all other groups, and Benders had higher JH titers than nB females and workers. For all models, Colony had no effect.

Queens consistently showed higher JH titers than other female types assayed alongside (Additional file 1: Figure S2 (blue axes)). In a pooled analysis including data from all queenright colony conditions, queens had significantly higher JH titers than newly emerged females (<24 hours since eclosion), 1–3 day old pre-workers and workers, workers ≥4 days old, and young attacked benders from colony 10 (see Figure 3A). To determine whether JH titers correlated with reproductive dominance, we recorded oviposition events over an 8-day period (8 h/day) in colony 5 when cells became vacant because of adult emergence in addition to egg removal by the observer. Queens (N = 28), some of which were bled after the observations concluded, were observed to oviposit 0–8 times. There was no difference in JH titer between queens with low (0–2 ovipostion events; N = 6) and high (6–8 oviposition events; N = 5) short-term fecundity (two tailed *t*-test, t = 0.49; DF = 9; P = 0.62; Z_r_ = 0.076) (Additional file 1: Figure S2C). Also, there was no significant correlation between ovary size (number of oocytes >70% LME) and JH titers among queens sampled (colony 4: N = 4, Pearson’s *r* = 0.2, P = 0.80, Zr = 0.08; colony 5: N = 11, *r* = −0.45, P = 0.18, Zr = 0.28; colony 6: N = 12, *r* = 0.57, P = 0.08, Zr = 0.64; colony 7: N = 7, *r* = −0.46, P = 0.30, Zr = 0.11; colony 10: N = 4, *r* = 0.8, P = 0.2, Zr = 0.09).

Queenright females aged 1–3 adult days, which include pre-workers and young workers, had higher JH titers than workers over the age of 3 (Figure 3A), although this pattern was not seen in all colonies (e.g., Additional file 1: Figure S2). Among workers from colonies 4–10 where most of the envelope was removed (e.g., Figure 1C), JH titers were very low (Figure 3 and Additional file 1: Figure S2). In colonies where collected foragers had a ‘cuticular age’ score higher than builders (two-tailed *t*-test on cuticular age: colony 9: t = 3.00, DF = 6, P = 0.024; colony 10: t = 4.87, DF = 13, P = 0.0003) or did not (colony 4), JH titers were nearly identical between the builders and foragers (means within 1 pg/μl). Yet in colonies with only a small piece of the envelope removed (e.g., Figure 1B), a wider range of JH titers was observed, with nectar foragers having the highest of all (Additional file 2: Figure S3).

### Effects of queen removal

Females that engaged in bending displays for 1–3 days following queen removal (referred to as ‘new benders’) consistently had higher JH titers than comparably aged workers before queen removal (Additional file 1: Figure S2 and Additional file 3: Figure S4A) as well as workers that continued to work in the absence of queens (Additional file 1: Figure S2 (red axes)). As mentioned, new benders could be comprised of former workers, younger idle females, and/or females that emerged in the absence of queen (Table 1). In a pooled analysis including data from queenless nests, benders that had worked prior to queen removal had higher JH titers than benders without a history of working, and both types of benders had higher JH titers than newly eclosed wasps and workers (Figure 3B). Finally, statistical analyses revealed no significant correlation between JH titer and oocyte length among these new benders (e.g., colony 5: N = 15, Pearson’s *r* = 0.15, P = 0.59, Z_r_ = 0.09; colony 6: N = 20, Spearman’s ρ = −0.05, P = 0.83, Z_r_ = 0.14).

In colonies 6 and 7, we allowed a set of ‘new benders’ to become established for one week (Additional file 1: Figure S2D-E (green x-axes)). In both colonies, the JH titers of these ‘established benders’ were highly variable and showed no statistical correlation with oocyte length (colony 6: N = 5, Pearson’s *r* = −0.41, P = 0.49, Z_r_ = 0.064; colony 7: N = 6, Pearson’s *r* = 0.47, P = 0.35, Z_r_ = 0.34). In colony 6, one bender was being chased and attacked by a worker, and a former bender was observed building. These ‘defeated benders’ had baseline JH titers. In colony 7 (Additional file 1: Figure S2E), the established benders were comprised of 4–8 day old adults that had eclosed after queen elimination. Females aged 1–4 days did not bend, frequently huddled (i.e., remained idle) instead of working and were sometimes attacked. These suppressed ‘huddlers’ had very low JH titers (Additional file 1: Figures S2E and Additional file 3: S4B). In a pooled analysis that included data from both of these colonies, the established benders had higher JH titers than the suppressed females (the attacked bender of colony 6 and huddlers of colony 7), newly emerged females and their worker counterparts (Figure 3C).

When data for all females from queenright and queenless conditions were combined, both queens and benders (encompassing all pre-reproductive bending subtypes) had higher JH titers than 0–3 day old non-benders and workers ≥3 days of adult age (Figure 3D).

In colony 10, the removal of 3 of 4 queens (single queen reign is indicated by the light blue x-axis bar in Additional file 1: Figure S2G) led to the emergence of young benders in queenright conditions (see above). Among the benders, attacking workers and queen, only the latter had an elevated JH titer (Additional file 1: Figure S2G and Additional file 4: Figure S5). Once the last queen was removed, JH titers were clearly elevated in the three queenless benders (2–5 day old adults) on the following day (Additional file 1: Figure S2G (red axis) and Additional file 4: Figure S5).

### Correlation with oocyte length

JH titers were positively correlated with oocyte length in all six colonies where ovaries were measured (Spearman’s ρ for each nest: P < 0.004) (Additional file 5: Figure S6). This correlation was counteracted to some extent by the higher than expected JH titers (based on oocyte length) of new benders and the lower than expected titers of suppressed ‘hopeful reproductives’ (e.g., huddling and/or attacked non-workers). These patterns, along with the lack of statistical correlation between oocyte length and JH titer within bending groups (see above), suggest that JH titers are more associated with the activation of the ovaries than with basal oocyte size.

### Ecdysteroids

**Figure 4:**
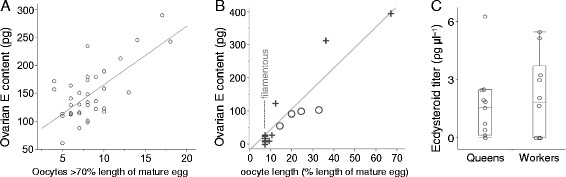
**Ecdysteroids levels in ovaries and hemolymph.** Ovarian ecdysteroid contents correlate positively with: **(A)** ovary size (number of oocytes >70% length of mature egg) in queens from colonies 6–10 (N = 36, Spearman’s ρ = 0.51, P = 0.0014), and **(B)** oocyte length in workers (+) and benders (o) from colony 4 (N = 15; Spearman’s ρ = 0.87; P <0.0001). **(C)** Hemolymph ecdysteroid titers did not statistically differ between queens and workers in colony 8 (Kruskal-Wallis test: N = 21, H_1_ = 0.18, P = 0.86, Z_r_ = 0.075). Box plots show the median and the middle two quartiles; the whiskers indicate the 1.5 interquartile range.

Ovarian ecdysteroids were correlated with queen ovary size (number of oocytes >70% LME) (Figure 4A). Among females where oviposition was not observed, ovarian ecdysteroids clearly correlated with oocyte length in pooled workers and benders from colony 4 (Figure 4B).

Hemolymph ecdysteroids were measured in two colonies. Queens and workers of colony 8 showed no difference in ecdysteroid hemolymph titers (Figure 4C). Also, only 3 of 13 samples from colony 4 had detectable amounts, none of which came from queens (N = 4).

### Effects of exogenous JH

In experiments where we were interested to see whether JH may affect behavior we used JH III, the natural JH of Hymenoptera [43-45], and methoprene, a JH mimic. In most insects, including bees [46], the natural JH is rapidly metabolized, and so if a change in behavior (e.g., aggression) was not observed within 24 hours of JH III application, we repeated the treatment for a total of three consecutive days. By contrast, in experiments where we were interested to find out whether JH may have a gonadotropic effect or provoke an alteration in the cuticular hydrocarbon pattern in these wasps we used methoprene. This JH mimic is a more stable molecule than JH III as it is not degraded as efficiently by JH esterase [47]. It is therefore better suited for investigating longer lasting effects and requires fewer applications.

On a nest containing brood of all ages, all queens and workers <3 weeks of adult age were removed (colony 8). Forty-six of the ~55 remaining workers received a dose of 10 μg of JH III (N = 23) in 1 μl of cyclohexane or just 1 μl of cyclohexane (N = 23) per day over three consecutive days starting with the day of removal of the last queen. The nest produced no adults during the first two days of treatment, and two newly emerged females were removed when they appeared on the third and fourth days. No bending or queen-like behavior was observed among any of the treated or non-treated workers.

**Figure 5:**
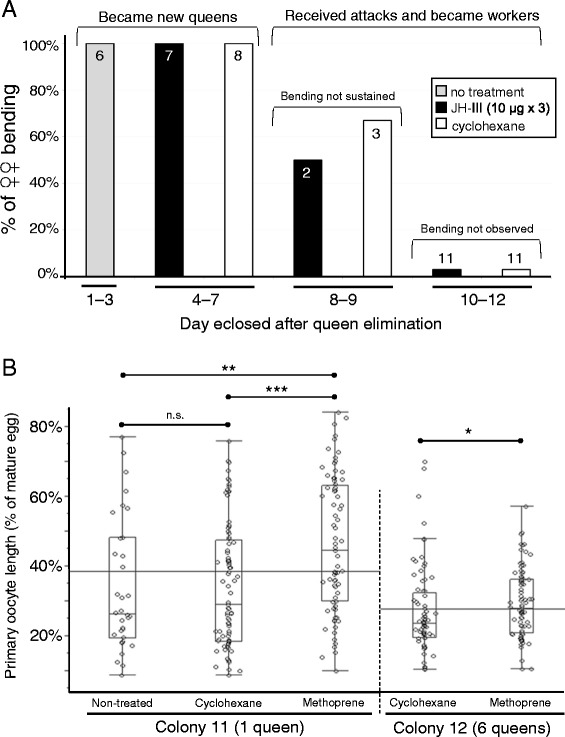
**Hormone manipulation experiments. (A)** JH III treatment of young queenless workers. JH III treated females showed no difference in bending propensity compared to cyclohexane-treated controls. **(B)** Effect of methoprene treatment on oocyte development in young queenright females from colonies 11 and 12. Each data point represents 1 of a possible 6 measurable primary oocytes from treated and non-treated females (the horizontal line represents the grand mean shown for each nest). In colony 11, methoprene-treated females had longer oocytes than both control types (Steel-Dwass all pairs: vs. non-treated: N = 111, *Z* = 3.25, P = 0.003 = **; vs. cyclohexane: N = 166, *Z* = 4, P = 0.0002 = ***). In colony 12, where a comparison was made with only a solvent control, the difference was less obvious but still significant (Mann–Whitney U test: N = 165, *Z* = 2.12, P = 0.03 = *).

After finding no behavioral effect upon JH III treatment of old workers of colony 8, treatments were redirected toward newly emerged queenless females (one treatment per day for the first three days after eclosion). The first six females to eclose on the queenless nest assumed bending and were not treated. The following 15 females to emerge joined the cohort of future queens, irrespective of whether or not they had received JH III. All females to emerge thereafter were attacked and became workers, and JH III treatment had no ostensible effect on their bending behavior (Figure 5A). Thus, JH III treatment failed to induce the dominance behaviors frequently displayed by females with uncertain reproductive fates.

### Methoprene effects on behavior and ovarian development

To determine the effect of exogenous JH on ovarian development, young workers from queenright colonies 11 and 12 were treated twice (at adult age of 3 and 6 days) with methoprene or cyclohexane as a solvent control. Colony 11 also included non-treated wasps that were anesthetized alongside the treated females. Each female had begun building before the initial treatment. By the adult age of 4 days on colony 11, all methoprene-treated females began to receive one-on-one biting attacks from older workers that contacted them. The methoprene-treated females were mounted and bitten over large portions of their body (e.g., head, thorax, abdomen and/or appendages) including the face which induced an offering of liquid to the attacker or at least a gesture of it. The methoprene-treated female, having endured the attacks motionless in a crouched or slightly rolled over to the side position, would sometimes dash away when the aggression waned or the attacker moved on. This interaction strongly resembles the stereotyped, suppressive caste-determining attacks often received by young idle females in nests of *Synoeca* (and was observed in colony 11 when the nest envelope was first removed) and potential reproductives of related genera [2,4]. By days 7–9, the attacks had increased in severity and the attackers were, apparently, no longer appeased by subordinate gestures, compelling the attacked methoprene-treated wasps to flee towards isolated areas on or off the nest; by day 10 the first of the methoprene-treated females had disappeared (4 of 4). Ovarian measurements and cuticular hydrocarbon extractions (see below) were therefore done on females at the adult age of 7 days. Young workers from colonies 11 and 12 were monitored and treated at the same time. Observations were restricted to colony 11 (1–2 hours/day for 17 of 18 days), although attacks were also observed in colony 12 (N = 4) when females were collected for treatment. In both nests, only methoprene-treated wasps were observed to be attacked; in colony 11, 100% of the methoprene-treated wasps (N = 17) were attacked.

For this experiment, all six primary oocytes in the two ovaries (three ovarioles each) were measured to provide an in-depth description of the ovarian status. When evaluating separately the length of each primary oocyte (i.e., each set of ovaries potentially contributing six data points), methoprene-treated females had significantly longer oocytes than cyclohexane and non-treated controls for monogynic colony 11 and, to a lesser but also significant extent, polygynic colony 12 (Figure 5B), thus implicating JH as a gonadotropin.

### Cuticular hydrocarbons (CHCs)

An analysis of worker CHCs identified 22 compounds (Additional file 6: Table S1 and Additional file 7: Table S2) which appeared in every sample at detectable amounts. Identified compounds were either alkenes or linear alkanes.

### Queens vs. workers

**Figure 6:**
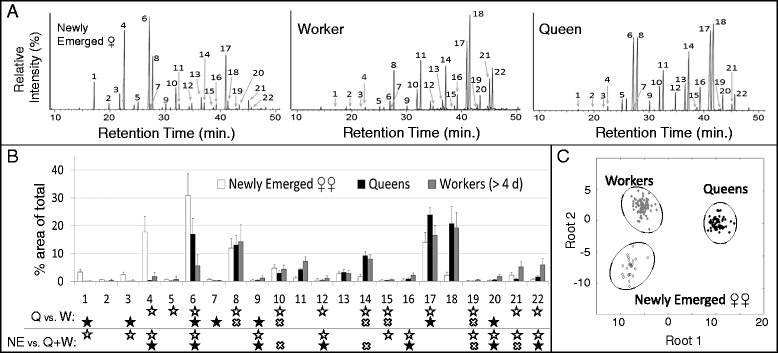
**Cuticular hydrocarbon (CHC) profiles of** ***Synoeca surinama*** **. (A)** Representative chromatograms of whole-body extracts for main female types*.* For compound numbering and identification see Additional file 6: Table S1. **(B)** CHC profiles of newly emerged females (NE), queens (Q) and workers (>4 days since eclosion) (W) from colonies 4–8 (mean % ± SD). Discriminant analyses were performed for queens vs. workers (w/out PCA: Global Wilks’ λ =0.01489; F_16,145_ = 599.68; p < 0.001; w/ PCA: Global Wilks’ λ = 0.03855; F_11,150_ = 340.06; p < 0.001), and newly emerged females vs. pooled queens + workers (w/out PCA: Global Wilks’ λ = 0.00148; F_34,334_ = 245.96; P < 0.001; w/ PCA: Global Wilks’ λ = 0.15546; F_15,170_ = 61.570; p < 0.001). White and black stars indicate the main contributors of separation prior to and following PCA, respectively. “X” denotes a compound removed based on the PCA analysis. **(C)** Canonical scatterplot based on a discriminatory analysis for all three groups.

The CHC profiles of queens (N = 67) and workers over the age of 4 days (N = 95) from colonies 4–8 were compared. A representative chromatogram for each phenotype is shown in Figure 6A. Adult females of 4 days and younger were excluded from this analysis because younger females experience a major age-related change in their CHC profile (see below). A stepwise discriminant function analysis (DA) based on all 22 shared compounds significantly separated individuals according to their status. A Principal Components Analysis (PCA) reduced the variable (i.e., CHC) number to 16, and the DA based on these compounds produced similarly robust results (see Figure 6B for statistics). The C25:1 monoene, 9-Pentacosane (CHC ID number 6 in Figure 6), was the only compound that was significantly higher in queens (but not in newly emerged females; see below) across all colonies (the C31:1 monoene, CHC ID number 17, was higher in queens in most but not all colonies examined). Workers tended to have a higher proportion of longer hydrocarbons (e.g., C32, C33:1, and C33).

### Newly emerged vs. older females

Newly emerged females (<24 h since eclosion) had a very different CHC profile than older females (Figure 6A-C). A DA based on all compounds shared between newly emerged females (N = 24) and queens + workers (N = 162) significantly separated individuals according to their age. The PCA reduced the variable (i.e., CHC) number to 17, and the DA based on these compounds also separated the groups, although the difference was less marked (see Figure 6B for statistics). A canonical plot of a DA performed for all queens, workers and newly emerged females is shown in Figure 6C.

### The emergence of caste-specific profiles

**Figure 7:**
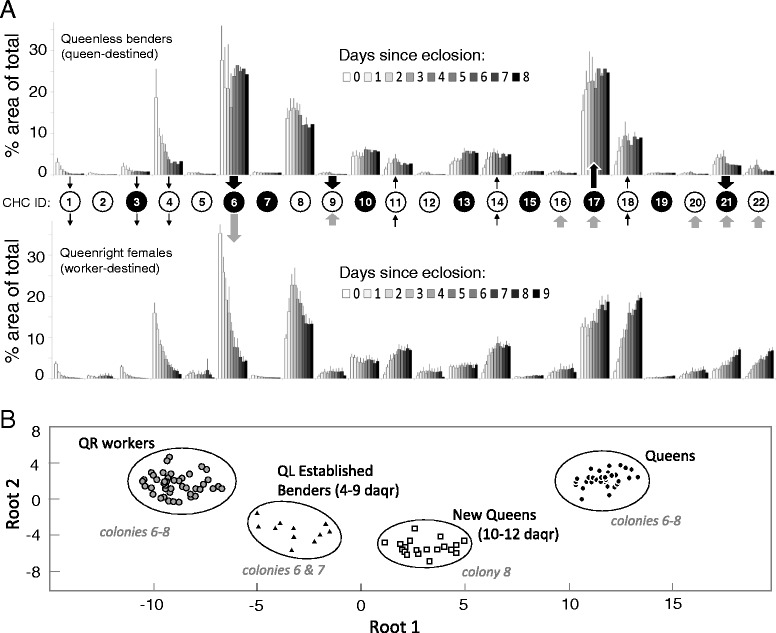
**Age and status related changes in cuticular hydrocarbon (CHC) profiles of** ***Synoeca surinama*** **. (A)** Relative percentages of CHC compounds in females in queenless (top) and queenright (bottom) conditions from colonies 5 and 7 (mean % ± SD). Linear alkanes are shown in white circles; alkenes are shown in black circles (for identities, see Additional file 6: Table S1). The surrounding arrows indicate evident changes in a compound’s proportional representation over the first 8 or 9 days of adult life and also show a difference between queen and workers (see Figure 6). Small arrows specify an age-specific shift, thick black arrows indicate a queen-like shift and thick gray arrows signify a worker-like shift. **(B)** Canonical scatterplot based on a discriminant analysis of CHC profiles for females belonging to colonies 6–8. daqr = days after queen removal.

The CHC profile of newly emerged females became more worker- or queen-like depending on the social context (Figure 7A). For all maturing females, shorter CHCs (e.g., C21 and C23 alkanes, and C23:1 monoene) decreased in proportion while longer ones (C27, C29, C31:1, C31) increased. In queenright females, C25:1 decreased while larger compounds, such as C32, C33:1 and C33, increased in proportion. Maturing queenless benders showed just the opposite pattern, marking the emergence of a queen-like CHC profile. In support of this, a DA for colony 7, where both 4–9 day old queenright workers and queenless benders were sampled, showed that benders have a CHC profile intermediate between workers and queens (Additional file 8: Figure S7A).

The development of a queen-like CHC profile was also evident in old workers that made the transition to bending (colony 6). After a day of displaying, these females did not show a distinct CHC profile from their worker counterparts sacrificed alongside or on the days prior (during queenright condition) (Additional file 8: Figure S7B and Additional file 6: Table S1). A week after queen removal, both workers and established benders showed a change in their CHC profiles, with benders showing a queen-directed shift (Additional file 8: Figure S7B). The two failed benders from this colony (see above) grouped according to their behavior at the time of collection: a former bender that was observed working had a queenless worker profile whereas the active bender that was chased and attacked grouped with the cohort of established, accepted benders (Additional file 8: Figure S7B).

For colony 8, established queens and, subsequently, young replacements queens (10–12 days old) were sacrificed. Unlike benders sampled from other colonies, these benders had begun laying eggs, and the majority of them were observed to fly off the nest near dusk, perhaps in search of mating opportunities. A DA based on all 22 compounds (PCA identified all compounds as contributors) was performed for queenright workers (>4 days old), queens and established benders from colonies 6–8. As expected, these new queens exhibited a CHC profile that was intermediate to established benders (i.e., pre-queens) and established queens (Figure 7B).

### CHC profile of methoprene-treated queenright females

**Figure 8:**
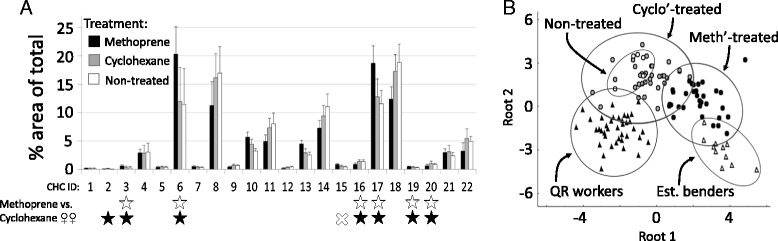
**Effect of the methoprene on the cuticular hydrocarbon (CHC) profile of** ***Synoeca surinama.*** **(A)** Methoprene application induced a CHC profile similar to that of incipient reproductives. The CHC profile of methoprene-treated females was distinct from cyclohexane- and non-treated females (pooled from colonies 11 and 12). For compound identification, see Additional file 6: Table S1. White and black stars indicate the main contributors of separation prior to and following PCA, respectively. “X” denotes a compound removed based on the PCA analysis. **(B)** Canonical scatterplot based on a discriminatory analysis for all treatment groups from colonies 11 and 12, including established benders and queenright workers from colonies 6 and 7 showing a clearly significant difference, done with and without PCA (for both: Global Wilks’ λ = 0.185; F_13,51_ = 17.3; p <0.001; 100% predicted classification). The non-treated controls group within the cyclohexane-treated wasps whereas methoprene-treated females overlap with the established benders, but not the queenright workers.

The CHC profiles of the attacked methoprene-treated workers from colonies 11 and 12 (see above) were distinct from their cyclohexane and non-treated counterparts (Figure 8 and Additional file 7: Table S2). Methoprene-treated females possessed an established bender-like CHC profile, while non-treated females were nested within the cyclohexane-treated workers (Figure 8B). Importantly, when females from each nest were split into two groups according to oocyte length, irrespective of treatment, the differences, although significant, were notably less so (colony 11: Global Wilks’ λ = 0.650; F_5,37_ = 4.0; P < 0.001; 81% classification; colony 12: Global Wilks’ λ = 0.182; F = 25.4; P < 0.001; 95% classification). Therefore, methoprene application appears to affect the CHC profiles of young females independently of its gonadotropic role.

## Discussion

To our knowledge, this is the first study to couple endogenous endocrine measurements and hormone manipulation experiments for a permanently eusocial wasp. For *Synoeca surinama*, we show that JH titers are consistently higher in incipient and actual queens than in workers, suppressed ‘hopeful reproductives’ and newly emerged females (Figure 3 and Additional file 1: Figure S2). This pattern suggests that JH has conserved gonadotropic, behavioral and/or chemical signaling functions that these wasps share with their non-swarming primitively eusocial relatives (e.g., *Polistes*) [27,48,49]. Yet in further hormone assays combined with subsequent JH and methoprene application experiments for *S. surinama*, it became evident that JH is not required for the expression of aggressive displays and failed to influence the caste trajectory of females with uncertain fates. Instead, JH appears to both promote ovarian development and direct a shift in the CHC profile toward that of a replacement queen. This is in contrast to another caste-flexible epiponine wasp, *Polybia micans*, where high JH titers are not typically sustained in maturing reproductives [34]. One shared aspect in the endocrinology of *S. surinama* and *P. micans* is the apparent lack of ecdysteroid function in the hemolymph, which in *Polistes* is important for establishing dominance [25,28,29].

### JH and behavior in *S. surinama*

In *S. surinama*, queens from each colony had higher JH titers than other types of females (Figure 3A,D) while accepted queenless benders had more than their worker or idle counterparts that did not display (Figure 3B,C and S2). Nonetheless, JH titers were highly variable in these cohorts of accepted queenless benders, with some females having no detectable amounts. Without exception, all queenright and queenless benders which were suppressed through physical aggression had very low JH titers (Figure 3A,C). What remains to be explained is why queenless females which transitioned from working to bending had higher JH titers than bending cohorts which had not previously worked (Figure 3B).

These data show that elevated JH levels are not required for the expression of aggressive displaying in *S. surinama*, but these assays could not test whether JH plays a role in augmenting aggression. To address this, we showed that JH-III treatment failed to induce bending in old workers on a nest lacking both queens and young females. Despite their putative totipotency (cf. old workers-turned-benders in colony 6), all workers, treated or not, forewent the opportunity for direct reproduction on a nest where new adults were about to emerge. JH-III treatment also had no effect on young females that had eclosed in a period where an individual’s caste fate was less restricted due to lack of queen(s) (Figure 5A). One caveat to these results, which showed that there was no obvious behavioral response in treated females, is that the manipulations lacked a positive control, i.e. a treatment with a JH mimic that is more stable than JH III. Thus, we cannot rule out the possibility that exogenous JH-III were counteracted to some extent by JH-degrading enzymes, most probably a JH esterase (JHE), as shown in honey bees [50], where JH-III treatment strongly induced JHE expression. That said, we were deliberately testing for a trigger effect of JH-III on aggressive behaviors, and it is likely that the exogenous JH-III was present for at least a few hours to a day before it could be fully degraded. Thus, we conclude that JH is not a direct activator of aggressive displays in females with open fates in *S. surinama*, a result completely at odds with other social wasps, where a boost in JH is thought to enhance competitive ability in unstable circumstances. For example, in the fellow epiponine *P. micans*, JH levels were seen to spike in potential reproductives that may become queens [34]. In *Polistes*, where physical aggression is the common expression of dominance [51], JH levels are higher in winners among competing reproductives [48,49,52], and are particularly high in workers that compete to replace a fallen queen [26].

A link between dominance and JH titers might not become established until incipient reproductives mate and become queens, where competition is high, egg-guarding vigils initiate and ritualized aggression is common [1,2,6]. Although no dominance hierarchy appears to be operational in *S. surinama*, inequalities among queens are likely to exist. Nonetheless, among queens, JH titers did not significantly correlate with large oocyte number in any colony, and when comparing queens with relatively high and low oviposition rates, there was no difference in JH titers (Additional file 1: Figure S2C), suggesting that JH is not directly related to dominance or fecundity within a cohort of queens. Yet because there were always a few brood cells empty in the aforementioned nest, this may have encouraged egg-laying among queens while reducing dominance expressed by way of differential oophagy, a behavior highly correlated with dominance in wasps [10]. In terms of direct displays of dominance, it is noteworthy that the one queen observed to physically attack other benders had the highest JH titer in the colony (Additional file 1: Figure S2G), suggesting that in some cases JH may elevate during periods of intense competition. Overall, however, *S. surinama* stands alone among the currently studied social wasps in providing evidence that JH is possibly only of secondary importance for behavioral signals of dominance (i.e., aggression) in the context of reproductive competition.

Other candidate drivers of aggression are the ecdysteroids. In *Polistes*, the ovaries release ecdysteroids into the hemolymph [28], and injection of 20E increases a foundress’ chance of becoming dominant (similar to JH) [25]. Yet in *S. surinama* the hemolymph ecdysteroid levels were low and indistinguishable between queens and workers (Figure 4C). Therefore, an unknown factor must incites aggressive displays in *S. surinama.* Biogenic amines, functioning as neurohormones, are good candidates given their role in reproduction [53,54,3], aggression [55,56] and arousal [57] in other eusocial Hymenoptera.

Although worker behavior in *S. surinama* was not studied herein in detail, queenright females in envelope-damaged nests clearly built before they transitioned to foraging, as observed by West-Eberhard [1]. In colonies where only pieces of the nest were removed, some old foragers had elevated titers (Additional file 2: Figure S3). The scatter of JH titer values may, thus, reflect a change in JH titers associated with task transition, as has been suggested in other eusocial wasps [26]. Yet we found that females that built and/or foraged in envelope-removed nests generally had low JH titers, and only appeared to be slightly elevated in 1–3 day old queenright females (Figure 3A), an age span concomitant (in this study) with the onset of building and slight ovarian growth (Figure 2). Also, methoprene treatment to young queenright builders of *S. surinama* led to an increase in ovarian growth and received aggression (see below) instead of precocious worker activity, contrary to reports of other caste-totipotent polistine wasps: *Polistes canadensis* [21], *P. dominula* [30], and in the epiponine *Polybia occidentalis* [33].

### JH acts to coordinate ovarian maturation and incipient queen-like CHC profile in *S. surinama*

Although JH was not directly linked to reproductive dominance, there are multiple lines of evidence suggesting that JH functions as a gonadotropin in *S. surinama*: 1) JH levels were increased in benders that were not physically challenged by nestmates (Figure 3B); 2) JH titers were correlated with oocyte length in all colonies (Additional file 5: Figure S6); 3) JH was higher in queenright 1–3 day old females than established workers (Figure 3A) as their oocytes increased in length in the few days following eclosion (e.g., Figure 2); and 4) methoprene application led to an increase in oocyte length in queenright workers (Figure 5B). In the latter experiment, methoprene application had a much stronger effect on workers in a single-queen nest, suggesting that chemical signals from queens can modulate the gonadotropic effects of exogenous methoprene.

Methoprene application to the aforementioned queenright workers also induced a queen-directed shift in their CHC profiles, consistent with the positive relationship between JH titers and the queen-like CHC profiles of queenless benders. The putative fertility information conveyed in their CHC profile probably led to their attack by nestmates, although it is possible that other chemical signals were co-affected (e.g., pheromones from the head [2]). The intermittent suppressive attacks were occasionally observed on nests with young females, indicating that the methoprene-treated wasps were not attacked based on a foreign odor, especially since treated females were not observed to be attacked on the day of initial treatment. As has been suggested in other wasps [2,4,9,20,58] and honey bees [59], the objective of such stereotyped aggressive interactions is likely to curb reproductive growth and/or stimulate working in prospective reproductives. In methoprene-treated workers of *S. surinama*, the CHC profile continued to change toward a reproductive-like profile despite the initial attacks. This persistence likely resulted in the unrestrained aggression which eventually led to their permanent eviction from the colony.

Does JH directly affect the CHC profile of *S. surinama*, or might JH stimulate ovarian development which in turn modifies the CHCs? Some solvent-treated females that were never attacked had ovaries that were larger than those of some of the methoprene-treated females that were always attacked. This suggests that signaling through an altered CHC profile is more important in this context than oocyte size. Also, grouping females according to treatment (methoprene vs. solvent) resulted in a much better separation by CHCs than by oocyte length in both colonies studied. And as mentioned, biting attacks were observed a day after treatment, scarcely affording sufficient time for significant ovarian growth in the attackees. These results suggest a direct role of JH in modulating the CHC profile.

### Age- and social context-related changes in cuticular hydrocarbon profiles

In social insects, CHCs convey important information regarding age, reproductive status, sex, nest membership and more [60]. In *S. surinama*, newly emerged females possessed high amounts of relatively small hydrocarbons that became drastically reduced over the first few days of adult life (Figure 7A), a pattern comparable to *P. micans* [34]. The CHC profile of these aging females also becomes more worker- or queen-like depending on the social context of the nest (Figure 7A). For example, a proportional increase in long chain hydrocarbons was only observed in females that became workers (Figure 7A). Of particular interest is the dynamic of 9-Pentacosene (C25:1), which showed both age and caste related changes. In the CHC profile of newly eclosed females, this alkene represents 40% of the total CHC blend. In workers it drops precipitously to ~5%, whereas in incipient queens (i.e., females which emerged in queenless conditions) it stays above 15% (Figure 6B; compound number 6 in Figure 7). It is possible, then, that the aggressive approaches of worker and queens toward newly emerged females and other queens are elicited by the high representation of C25:1, and whether a physical attack results or not may depend on both the colony state (e.g., queenright or queenless nest) and the condition of approached female (e.g., expressed through behavioral and/or other chemical signals). Nevertheless, the fact that no hydrocarbon was found to be consistently higher in queens than in both newly emerged females and workers suggest that the composite of the CHC profile of a queen is more important in keeping workers sterile than any given compound. In other eusocial hymenopterans, including vespine wasps, single saturated hydrocarbons – expressed at relatively high levels in queens for a given species – are sufficient to reduce oocyte length in workers [61]. Yet in *S. surinama* those saturated hydrocarbons that showed caste-related differences were always higher in workers (Additional file 6: Table S1, Figures 6, 7), and so it unlikely that this class of pheromones is involved in controlling nestmate reproduction in this species.

In both *S. surinama* and *P. micans*, prospective reproductives with well-developed ovaries exhibited a rise in hydrocarbons associated with queen status, as would be expected, although the queen-associated compounds differed between the species. Indeed, whereas a methyl-branched alkane appears to be important for *Polybia* [34,62], likely the most derived genus of Epiponini [31,32], they were not consistently detected in either *S. surinama* (Figure 6 and Additional file 6: Table S1) or another epiponine, *Parachartergus aztecus* [63], which are characterized by a high composition of alkenes. Indeed, the diversity of hydrocarbon signaling within the Epiponini – in apparent contrast to temperate vespine wasps [42,61] – suggests these putative pheromones may be subject to strong social selection between workers and queens [1,42].

### Concluding remarks

With respect to social evolution in wasps, *Polistes*, *Synoeca* and *Polybia* are likely to share a eusocial ancestor that was caste-totipotent [31,32], and so it was expected that a common endocrine ground plan may underlie caste plasticity in these wasps, with JH and the ecdysteroids playing important roles in competition and reproduction, as shown in *Polistes* [14,22,24,48]. Yet when summarizing the major findings for these three wasp genera (Table 2), the idea of a grand unifying pattern of hormonal regulation in social wasp adults appears untenable, for aside from the observed rise in JH titers in potential queens (and some evidence that JH is involved in the behavior transitions of workers), the endocrine profiles of these caste-flexible wasps have little in common. Indeed, social wasp endocrinology appears to be as labile as the plasticity it was thought to underlie. And whereas the endocrine profile of caste-flexible *Polybia micans* appears to align more closely with that of highly eusocial bees than other wasps studied to date [34], JH titers for *S. surinama* appear more bumble bee-like than either *P. micans* or *Polistes*. For example, JH is likely gonadotropic in both bumble bees [64] and *S. surinama*, and although JH titers are generally associated with reproductive dominance [64], JH or methoprene treatment does not increase dominance in queenright and queenless conditions in either species [65,66]. To our knowledge, a role for JH in modulating the CHC profile of bumble bees has not been explored. In addition to the species listed in Table 2, there is evidence that JH drives age-related changes in behavior in *Polybia occidentalis* [33] and functions as a gonadotropin in the primitively eusocial *Ropalidia marginata* [67], but hormone levels have not been measured in either case.

**Table 2:** **Summary of differences in the endocrinology of three genera of wasps**

**Hormone & effect**	***Synoeca surinama***	***Polybia micans***	***Polistes sp.***
JH titers rise in reproductive competitors in a queenless colony	Yes (Figure 3B; Additional file 1: Figure S2)	Yes [34]	Yes [26]
JH titers remain elevated in potential reproductives as they become queens	Yes (Figure 3; Additional file 1: Figure S2)	No [34]	Yes [26]
JH higher in queens than workers	Yes (Figure 3A)	No [34]	Yes [21]
JH higher in lone (vs. multiple) queens	Maybe (Additional file 1: Figure S2F,G)	Yes [34]	n/a
JH and/or methoprene induces aggressive behaviors in potential reproductives	No (Figure 5A)	Not tested	Yes [23-25] (but see [48])
High JH sustains ovarian development	Yes (Figure 5B)	No [34]	Yes [22,23,49]
JH involved in regulating the CHC profile of maturing reproductives	Yes (Figure 8)	Maybe [34] (does not sustain)	Yes [27] (correlated in *P. dominula*)
JH involved in age-related changes of behavior of workers	Maybe (Additional file 2: Figure S3)	Maybe (H.Kelstrup, unpubl.)	Probably [21,30]
Ecdysteroids present in the ovaries	Yes (Figure 4A,B)	Yes [34]	Yes [28]
Ecdysteroids present in the hemolymph and augment aggressive behaviors	No (Figure 4C)	Doubtful [34]	Yes [25,28]

If caste-plasticity is conserved among a group of eusocial animals, how and why would the original regulatory factors controlling caste fate change? The diversity and apparent susceptibility to modification of wasp endocrinology is a treasure trove for such evolutionary and developmental research, and the ability to study behavior and endocrinology *in situ* in the field is a major advantage [68]. Fortunately, there are over 225 species from roughly 20 genera of Epiponini, facilitating the phylogenetic tracking of CHC signaling evolution and its effectors. Finally, this physiological work should keep an eye on future genomic studies, which in wasps [69], as well as ants and bees [70], have revealed lineage-specific changes not just in gene composition but in the regions where hormones, their receptors and co-activators operate: the regulatory elements [70].

## Methods

### Wasps

All behavioral and physiological data were collected from *Synoeca surinama* [Hymenoptera: Vespidae: Epiponini] nests found on the campus of Universidade de Federal Sergipe (UFS), São Cristóvão, Sergipe, Brazil. All nests of *S. surinama* were studied *in situ*, anywhere from 1 to 12 meters off the ground. Although nests can grow quite large (Figure 1D), for queen removal colonies we focused on relatively small nests where all females could be marked according to their activity within a couple days. Voucher specimens are preserved at Universidade de São Paulo (USP), Ribeirão Preto.

### Field studies

The paper nests of *Synoeca* consist of a vertical planar comb of cells and a brittle corrugated envelope (Figure 1). Envelopes were partially or completely removed to facilitate observations inside the nest [3,71]. The destruction of the nest envelope unavoidably induces a drastic change in task allocation toward restoring the envelope (e.g., building and pulp foraging). Thus, on some nests, only small pieces of envelope were removed (Figure 1B). On most nests, all females were marked with oil-based Sharpie pens based on their behavior (e.g., bending, building, etc.) or day of emergence from the brood cell. Queens and workers are easily distinguished based on behavior [3]. Queens, in addition to rarely working, walk much more slowly than workers, spend most of their time huddled with other queens in the corners of the nest, exhibit bending behavior toward both workers and fellow queens and receive the ‘queen dance’ from workers. In this study, all sampled females (N = 71) having this repertoire turned out to have large ovaries and opaque spermatheca, evidence of having mated. Young queenright adults, which are idle for several days following eclosion, also huddle in corners of the nest but do not exhibit bending behavior (except in rare cases as reported here), and after several days they transition to building in queenright colonies. Each colony was observed for at least 4 days to identify the general status of each female and the adult age of newly emerged ones. In colonies where all queens are removed, the above queen-repertoire is taken up by a subset of females that are referred to as “new benders”. Queenless benders that have displayed for a full week are called “established benders”, and their ascension to queenhood is considered complete when they begin laying eggs. When queen fecundity was assessed (colony 5), eggs were periodically removed from cells.

To avoid stress-related endocrine responses, no females were sacrificed until two days after the envelope was first removed. To limit the effect of possible circadian endocrine changes [72], all wasps were collected for processing from 13:00–17:00, placed in clean glass vials and buried in ice (0°C) within 15 s of removal. Collection almost always included multiple types of females, with the relevant pair determined by the objective of the assay (e.g., queens vs. workers).

Relative age of workers and queens was estimated by scoring (blind) the degree of apodeme and cuticular darkening on the 5^th^ gastral sternite [6,7,73]. A pictorial representation of this feature and the scores assigned are shown in Additional file 9: Figure S1 and is referred to as ‘cuticular age’.

### Collecting hemolymph for hormone measurements, cuticular hydrocarbon wash, and ovary measurements

Wasps were transferred on ice to the laboratory for processing; for details see [34]. All wasps were bled within 2 h since longer periods of cold anesthesia significantly affect JH titers in honeybees [74]. Two to 10 μl of hemolymph was withdrawn from between the anterior-most segments of the gaster with a microcapillary (Drummond Scientific Company, Broomall, PA, USA). Samples destined for JH measurement by radioimmunoassay (RIA) were transferred to 500 μl of acetonitrile. Samples destined for ecdysteroid measurement were preserved in 500 μl of methanol.

Subsequent to bleeding, cuticular hydrocarbons (CHCs) were extracted from females by placing them in 2 ml of hexane for 2–2.2 min. Hormone and CHC samples were kept at −20°C. The ovaries of these wasps were carefully removed in cold E & B Ringer solution (7.5 g NaCl and 0.35 g KCl/1 L distilled water) and photographed with a Leica EZ4D Microscope Camera. Ovaries were then placed in 500 μl of methanol for ecdysteroid measurement.

Queen ovaries were quantified by counting the number of oocytes >70% the length of a mature egg (LME). The two largest oocytes of females that were not queens were measured using ImageJ (NIH, Bethesda, MD, USA) and averaged. Oocyte length is given as a percentage of a LME, and oocytes <8% LME were considered filamentous.

### Hemolymph juvenile hormone titer analysis by radioimmunoassay (RIA)

JH was extracted from the haemolymph sample in acetonitrile following a liquid-phase separation protocol developed for honeybees [75]. For the radioimmunoassay, we used [10-^3^H (N)]-JH III (spec. activity 19.4 Ci/nmol, Perkin Elmer Life Sciences, Waltham, MA, USA), JH-III (Fluka, Munich, Germany), and a JH-specific antiserum [76] as detailed by [77] and [34]. JH titers of the samples were calculated by non-linear four-parameter regression on standard curve values (ImmunoAssay Calculations spreadsheet, Bachem, Bubendorf. Switzerland) and are expressed as JH-III equivalents (pg/μl hemolymph).

### Haemolymph titer and ovarian ecdysteroid content analysis by radioimmunoassay (RIA)

Haemolymph samples in 500 μl in methanol were cold centrifuged (4°C), and the supernatant transferred to RIA glass vials and dried by vacuum centrifugation. For the quantification of ovarian ecdysteroid content, interfering lipids were removed from the methanolic extract by reversed phase chromatography [38].

Ecdysteroids were quantified by RIA, as previously described [77,78] using an antiserum prepared against a hemisuccinate derivative of ecdysone [79,80] [23,24-^3^H (N)] ecdysone (Perkin Elmer) (NEN, spec. act. 102 Ci/mmol), and 20-hydroxyecdysone (20E; Sigma St. Louis, MO, USA). Details of this RIA protocol are in [77]. Results are expressed as 20E equivalents, calculated by the same regression analysis used for JH titers (see above), reported as pg/μl for the hemolymph samples, or as pg/ovary for the ovary samples.

### Cuticular Hydrocarbon (CHC) analysis

After evaporation of the wash hexane, the extract was re-suspended in 50 μl of hexane and 1 μl was injected into a combined gas chromatography-mass spectrometer (GCMS) (model QP2010, Shimadzu, Kyoto, Japan). Separation was achieved on a DB-5MS column of 30 m, with a helium gas carrier at 1.0 ml min^−1^. Oven temperature was initially set to 150°C, and ramped up 3°C min^−1^ until it reached 280°C and held for 20 min. Analyses were performed in the splitless mode. The mass spectra were obtained by 70 eV ionization. The chromatographs were analyzed with GCMS solutions (Shimadzu). CHCs were identified using authentic standards (linear alkanes) and/or by their molecular diagnostics ion. The positions of unsaturations in alkenes were identified according to the dimethyl disulfide derivatization technique [81] and analyzed with the same GCMS system mentioned above.

### Exogenous JH III and methoprene application

Females were treated with either JH III (Sigma) or methoprene (Zoecon, Palo Alto, CA). Both JH III and methoprene were dissolved in cyclohexane (HPLC grade, Sigma-Aldrich) at final concentrations of 10 μg/μl each and 1 μl was topically applied to the gaster of cold-anesthetized wasps. Control wasps received a solvent application, and in some cases, non-treated wasp controls were also included. To test for immediate behavioral responses, 10 μg of JH III was applied on three consecutive days to old (>3 weeks) queenless workers and, in another experiment, to 1–3 day old adult females who emerged as the colony was transitioning from being queenless to queenright. To assess JH effects on ovarian growth and chemical signaling, 10 μg of methoprene, which has longer-lasting effects than JH III [47], was applied to working wasps at 3 and 6 days following eclosion. All wasps were kept in isolation for 45–60 min before they were returned to the nest to reduce the spread of the topically applied hormone to nestmates. Unless otherwise noted, all nests containing treated females were observed for 1–2 hours each day before treatment or capture.

### Statistical analyses

The range of JH titers for a given nest was not always the same, attributable to the fact that the samples were collected and analyzed over a two year period. Also, low sample size for certain female types precluded statistical tests within individual colonies. Therefore, we looked for differences in pooled data sets by performing a Mixed Model analysis, the Restricted Maximum Likelihood Method, on software SPSS 21 (IBM, Armonk, NY, USA) and JMP 10.0 (SAS Corporation, Cary, NC, USA). Fixed factors included Status and, in a grand analysis, Colony Condition (i.e., queenright vs. queenless nest). Colony was designated as a Random factor. To look for relationships between variables within a colony or from samples run on the same RIA, a Pearson’s *r* (normal data) or Spearman’s ρ (non-normal data) test implemented in JMP10.0 was used for significance. When comparing two groups, a two-tailed *t*-test (normal data) or a Kruskal-Wallis rank sums test (non-normal data) was used. In the case of multiple comparisons, the Steel-Dwass method for all pairs was employed using JMP 10.0. In cases where no relationship was found between two variables, a power analysis was employed and reported as Fisher’s Refined Z (Zr) using JMP 10.0 and Statistica 12.0 (StatSoft, Tulsa, OK, USA).

### Cuticular hydrocarbon data

Principal components analysis (PCA) was used to define the main components to be compared, such that compounds missing in most individuals of a group, as well as compounds contributing less than 5% to the first two factors in PCA were excluded from the statistical analysis. The relative concentrations of the compounds used in the discriminant analysis were readjusted to 100% and each peak was transformed according to [82]. Following this, a stepwise discriminant function analysis was done to see if combinations of variables could be useful in predicting group. Wilks’ λ values were used to verify the individual contribution of each variable to the model. For details of the CHC profile analyses, see [34]. The statistical analyses were performed using the software Statistica 10.0.

## Additional files

Additional file 1: Figure S2. JH titers according to female status. JH titers across colonies 4–10. *Blue axis* indicates queenright (QR) conditions; *red axis* indicates queenless (QL) conditions within 3 days of queen removal; *green axis* indicates QL conditions 7–8 days after queen removal; *light blue axis* indicates QR conditions with 3 of 4 queens removed. Queens (Q) with relatively high (Q↑) and low (Q↓) oviposition rates (see text) are distinguished in colony 5 (C), as is the remaining lone queen (LQ) for colony 10 (G). Newly emerged (NE) females were collected <24 after eclosion. Workers (including worker-destined females) were split into two groups: pre-workers and new workers 1–3 days since eclosion (dse) (pW) and older workers (W). New benders (B), which had been observed to bend from 1–3 days, are separated according to their history: workers-turned-benders (W➔B), idle females-turned-benders (I➔B), females that were within their pupal case when the last queen was removed (P➔B) and newly emergent benders (NE/B). In some nests, workers attacked new benders (W➔Atk). As benders became established for 4 days or more (Est B), suppressed ‘hopeful reproductive’, such as defeated benders (Def B) or idle huddlers (I), were also present. Asterisks in C and E indicate groups that are shown in more detail in Additional file 2: Figure S3. Box plots show the median and the inner two quartiles; the whiskers indicate the 1.5 interquartile range. Sample size is also indicated. Additional file 2: Figure S3. JH titers in workers from envelope-intact colonies. Relative age and task performed by workers from colonies 1 and 2. The cuticular age is based on the score of apodeme and cuticular tanning of the 5th sternite (see Additional file 9: Figure S1). Additional file 3: Figure S4. JH titers in young queenright (QR) and queenless (QL) females. (A) Colony 5: QL females appear to have higher JH titers, although QR females showed a rise during the first few days following eclosion. QL females of adult age 1–3 were observed to bend, and when compared to 1–3 day old QR females, QL benders had higher JH titers than QR workers (two tailed *t*-test, t = 4.81, DF = 25, P = 0.0004). (B) Colony 7: QR females had very low to undetectable JH levels, whereas QL benders, fated to become the next queens, showed a scatter from low to relatively high titers. Younger QL females that emerged after the establishment of accepted benders were suppressed and had low JH titers. Additional file 4: Figure S5. In Colony 10, queens and queenless benders had significantly higher JH titers than pooled QR + QL workers (vs. Queens: *Z* = −3.06 P = 0.012; vs. QL benders: *Z* = −2.7). Queens had significantly more JH than QR benders (*Z* = −2.76) while QL benders tended to have higher JH titers than QR ones (*Z* = −2.45) (Steel-Dwass all pairs, P < 0.05). Box plots show the median and the inner two quartiles; the whiskers indicate the 1.5 interquartile range. Sample size is also indicated. Additional file 5: Figure S6. Correlation analysis of JH titers versus oocyte length. Among all females types, JH levels were positively correlated (Spearman’s ρ) with primary oocyte length (% length of fully mature egg) in (A) colony 4 (N = 35, ρ = 0.54, P = 0.0009), (B) colony 5 (N = 58, ρ = 0.43, P = 0.0008), (C) colony 6 (N = 80, ρ = 0.58, P < 0.0001), (D) colony 7 (N = 70, ρ = 0.48, P < 0.0001), (E) colony 9 (N = 53, ρ = 0.73, P < 0.0001), and (F) colony 10 (N = 43, ρ = 0.45, P = 0.004). Behavioral category along with queen state (QR = queenright, QL = queenless) is indicated in color. “New Benders” includes all females observed to bend within 3 days of queen removal; the “Suppressed ♀♀” category includes huddlers or bending females that received aggression. Additional file 6: Table S1. Mean percentage of composition and standard deviation (SD) of cuticular hydrocarbons in various types of females of *Synoeca surinama*. Colony (s) of origin noted on top. Ret. Time = Retention Time; dse = days since eclosion; QR = queenright; QL = queenless; D = days after queen removal. (PDF 396 kb) Additional file 7: Table S2. Mean percentage of composition and standard deviation (SD) of cuticular hydrocarbons in various types of methoprene-, cyclohexane- (cyclo) non-treated females from queenright colonies 11 and 12. Ret. Time = Retention Time; dse = days since eclosion. Additional file 8: Figure S7 Intracolonial alterations in the cuticular hydrocarbon (CHC) profile of emerging reproductives. The canonical scatterplots are based on discriminant analyses of the CHC profiles. (A) From colony 7: queens, queenright (QR) workers 4–9 days since eclosion (dse) and queenless (QL) established (Est.) benders of the same age. (B) From colony 6: queens, QR workers; QL workers and new benders 1–3 days after queen removal (daqr); and QL workers, benders and two defeated (Def.) benders 7 days after queen removal. All females from colony 6 had eclosed over a month prior. Circles encompass all profiles for a given group. Additional file 9: Figure S1. Subjective measure of relative age of individuals based on the analysis of apodeme and other cuticular darkening on the anterior portion of 5^th^ abdominal sternite. Representative samples: 1 = newly eclosed female; 2 = 7-day old builder; 3 = 12-day old worker; 4 = 20-day old forager; 5 = >35 day old forager.

## Abbreviations

CHC: Cuticular hydrocarbons
DA: Discriminate analysis
JH: Juvenile hormone
LME: Length of mature egg
