# Supplementary figures and images for: The role of juvenile hormone in dominance behavior, reproduction and cuticular pheromone signaling in the caste-flexible epiponine wasp, Synoeca surinama
Source: Front Zool. 2014 Oct 24;11:78. doi: 10.1186/s12983-014-0078-5 (PMC4219083; doi:10.1186/s12983-014-0078-5)

JH titers (pg/μl)

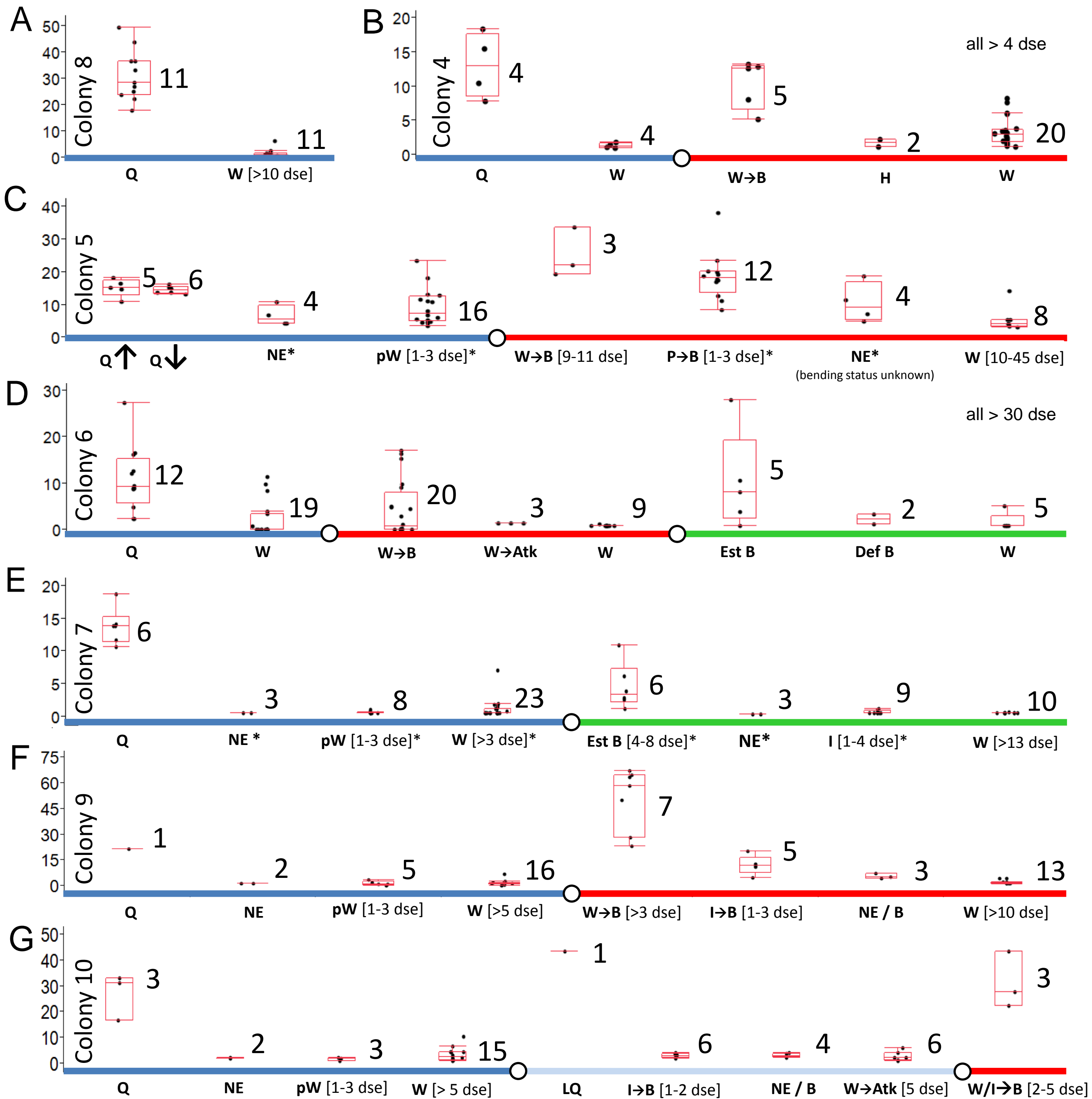

Supplement: Additional file 1: Figure S2. — JH titers according to female status. JH titers across colonies 4–10. Blue axis indicates queenright (QR) conditions; red axis indicates queenless (QL) conditions within 3 days of queen removal; green axis indicates QL conditions 7–8 days after queen removal; light blue axis indicates QR conditions with 3 of 4 queens removed. Queens (Q) with relatively high (Q↑) and low (Q↓) oviposition rates (see text) are distinguished in colony 5 (C), as is the remaining lone queen (LQ) for colony 10 (G). Newly emerged (NE) females were collected <24 after eclosion. Workers (including worker-destined females) were split into two groups: pre-workers and new workers 1–3 days since eclosion (dse) (pW) and older workers (W). New benders (B), which had been observed to bend from 1–3 days, are separated according to their history: workers-turned-benders (W➔B), idle females-turned-benders (I➔B), females that were within their pupal case when the last queen was removed (P➔B) and newly emergent benders (NE/B). In some nests, workers attacked new benders (W➔Atk). As benders became established for 4 days or more (Est B), suppressed ‘hopeful reproductive’, such as defeated benders (Def B) or idle huddlers (I), were also present. Asterisks in C and E indicate groups that are shown in more detail in Additional file 2: Figure S3. Box plots show the median and the inner two quartiles; the whiskers indicate the 1.5 interquartile range. Sample size is also indicated. [file 12983_2014_78_MOESM1_ESM.pdf]

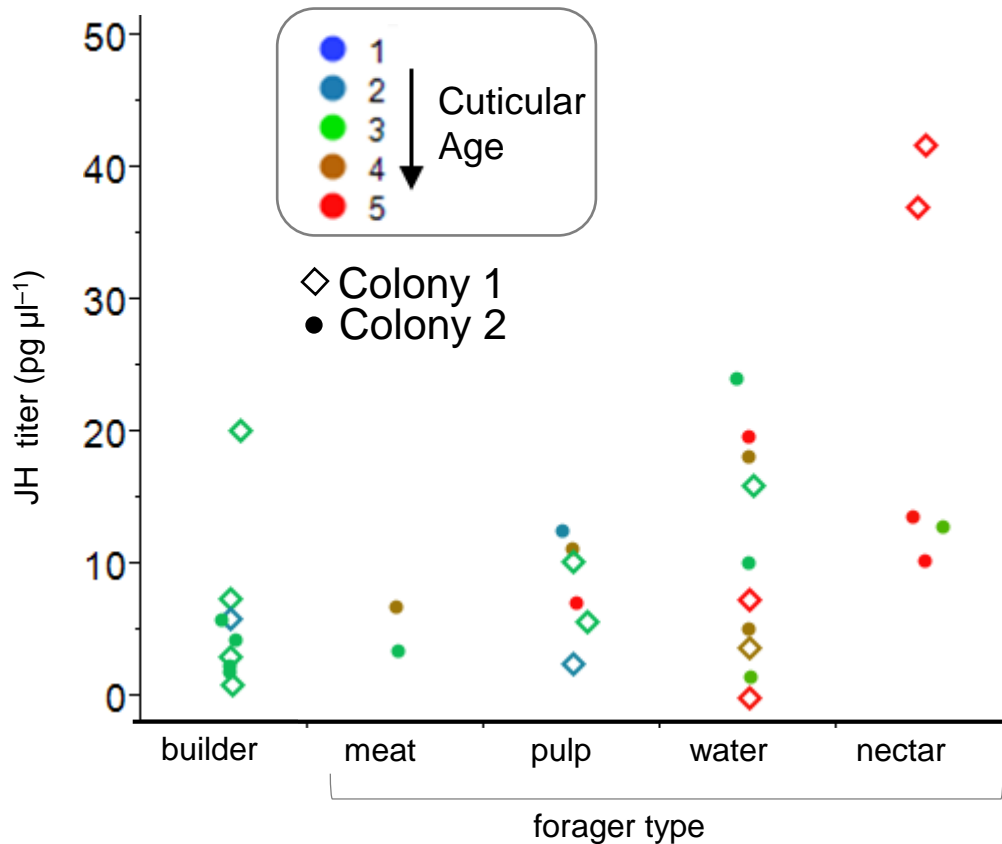

Supplement: Additional file 2: Figure S3. — JH titers in workers from envelope-intact colonies. Relative age and task performed by workers from colonies 1 and 2. The cuticular age is based on the score of apodeme and cuticular tanning of the 5th sternite (see Additional file 9: Figure S1). [file 12983_2014_78_MOESM2_ESM.pdf]

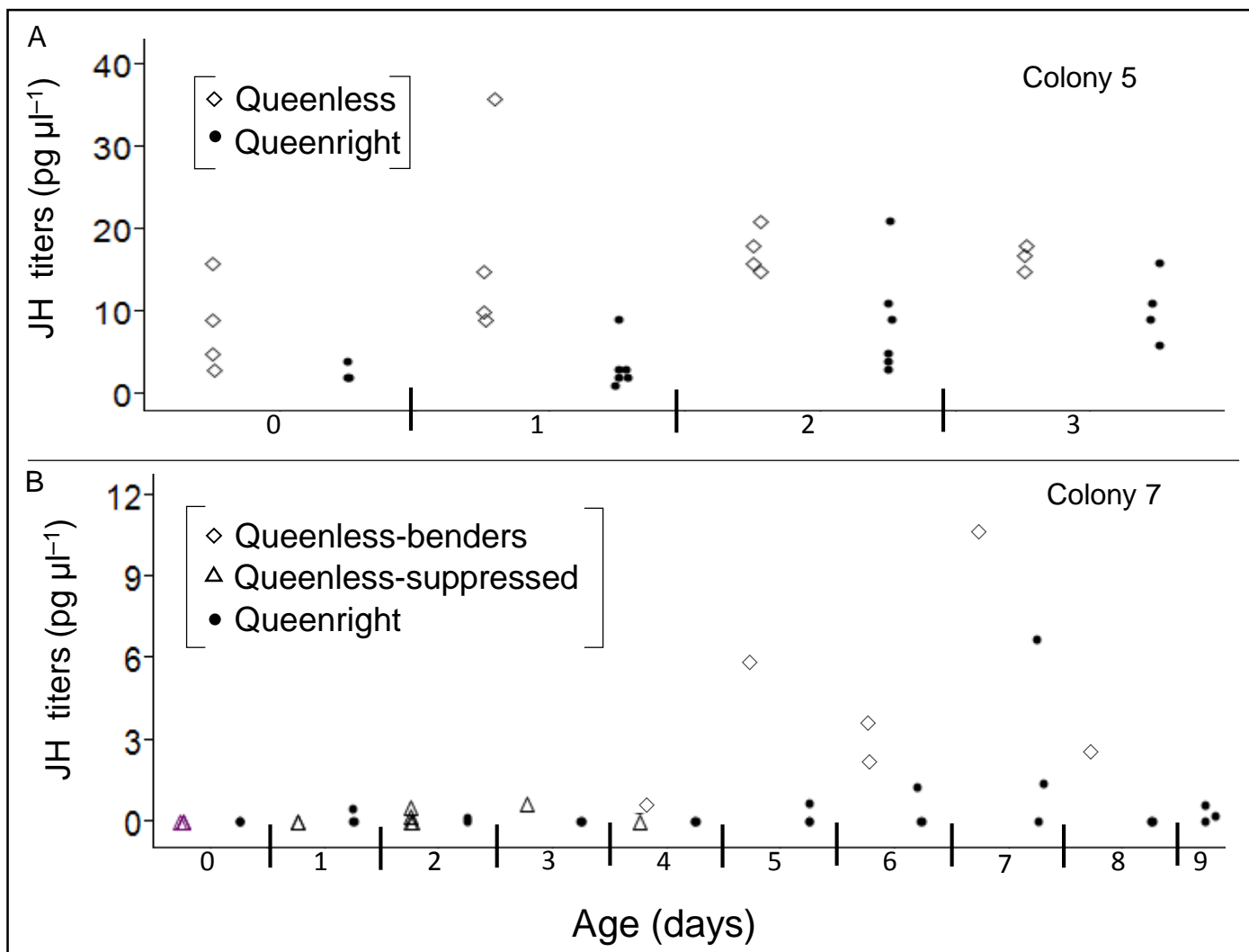

Supplement: Additional file 3: Figure S4. — JH titers in young queenright (QR) and queenless (QL) females. (A) Colony 5: QL females appear to have higher JH titers, although QR females showed a rise during the first few days following eclosion. QL females of adult age 1–3 were observed to bend, and when compared to 1–3 day old QR females, QL benders had higher JH titers than QR workers (two tailed t-test, t = 4.81, DF = 25, P = 0.0004). (B) Colony 7: QR females had very low to undetectable JH levels, whereas QL benders, fated to become the next queens, showed a scatter from low to relatively high titers. Younger QL females that emerged after the establishment of accepted benders were suppressed and had low JH titers. [file 12983_2014_78_MOESM3_ESM.pdf]

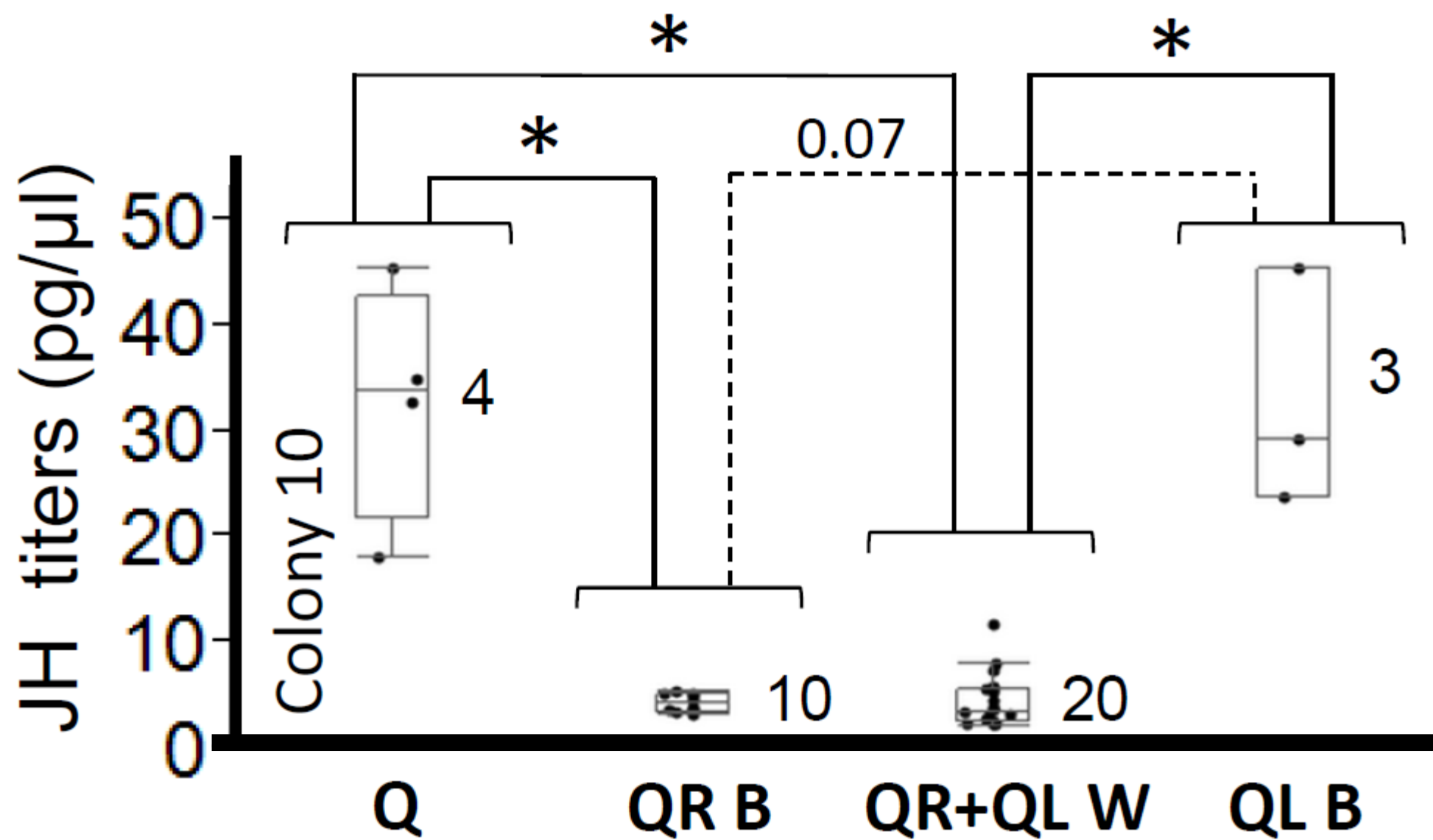

Supplement: Additional file 4: Figure S5. — In Colony 10, queens and queenless benders had significantly higher JH titers than pooled QR + QL workers (vs. Queens: Z = −3.06 P = 0.012; vs. QL benders: Z = −2.7). Queens had significantly more JH than QR benders (Z = −2.76) while QL benders tended to have higher JH titers than QR ones (Z = −2.45) (Steel-Dwass all pairs, P < 0.05). Box plots show the median and the inner two quartiles; the whiskers indicate the 1.5 interquartile range. Sample size is also indicated. [file 12983_2014_78_MOESM4_ESM.pdf]

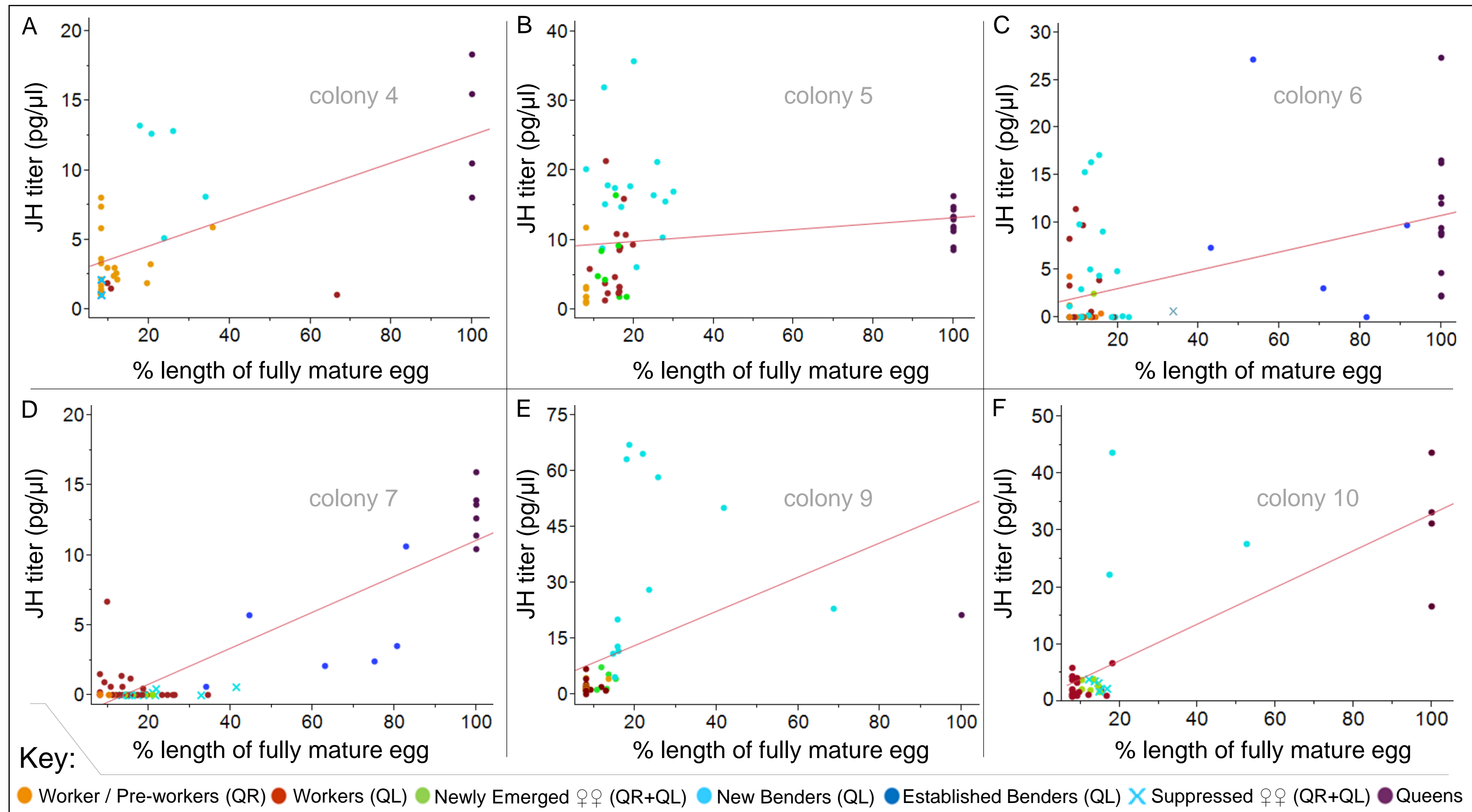

Supplement: Additional file 5: Figure S6. — Correlation analysis of JH titers versus oocyte length. Among all females types, JH levels were positively correlated (Spearman’s ρ) with primary oocyte length (% length of fully mature egg) in (A) colony 4 (N = 35, ρ = 0.54, P = 0.0009), (B) colony 5 (N = 58, ρ = 0.43, P = 0.0008), (C) colony 6 (N = 80, ρ = 0.58, P < 0.0001), (D) colony 7 (N = 70, ρ = 0.48, P < 0.0001), (E) colony 9 (N = 53, ρ = 0.73, P < 0.0001), and (F) colony 10 (N = 43, ρ = 0.45, P = 0.004). Behavioral category along with queen state (QR = queenright, QL = queenless) is indicated in color. “New Benders” includes all females observed to bend within 3 days of queen removal; the “Suppressed ♀♀” category includes huddlers or bending females that received aggression. [file 12983_2014_78_MOESM5_ESM.pdf]

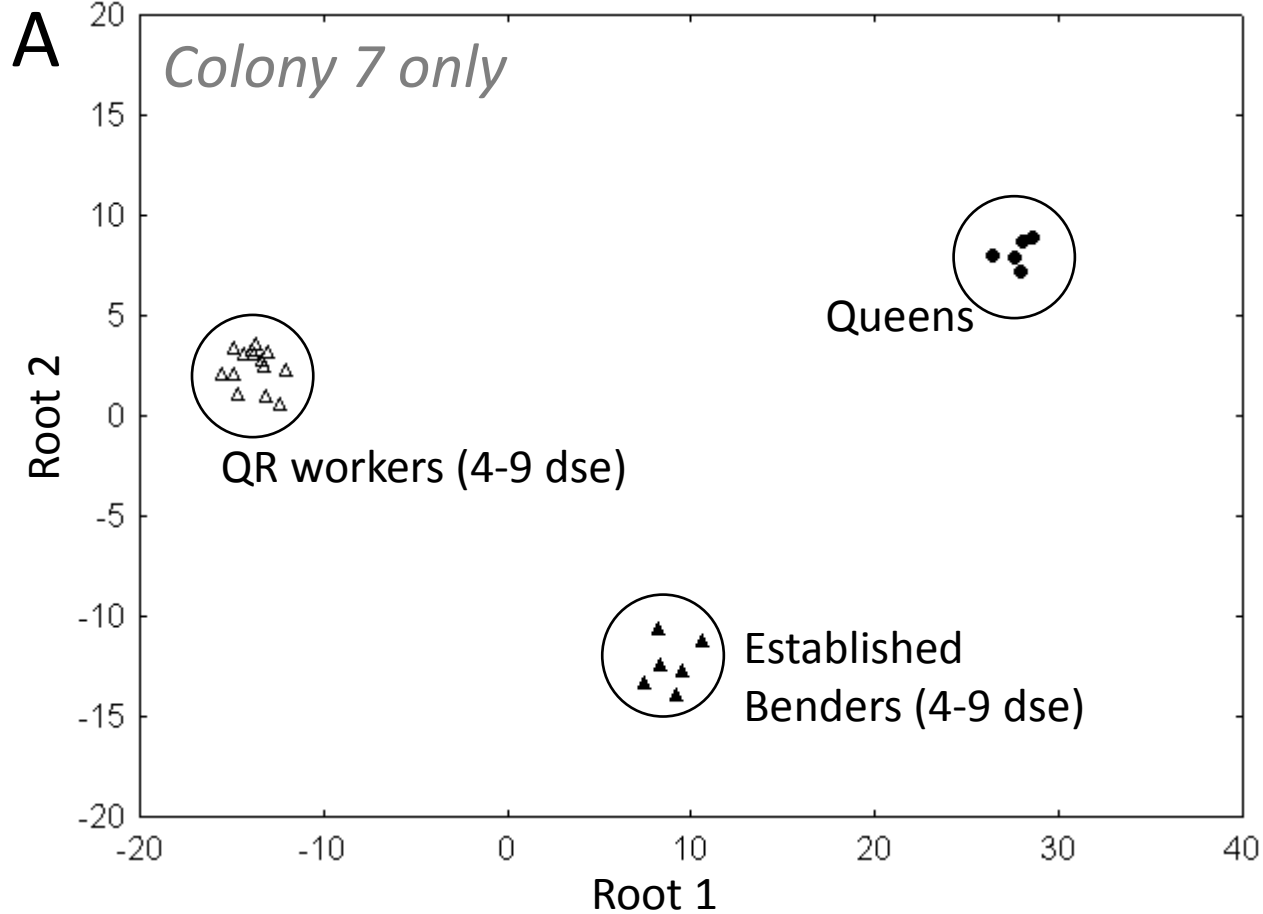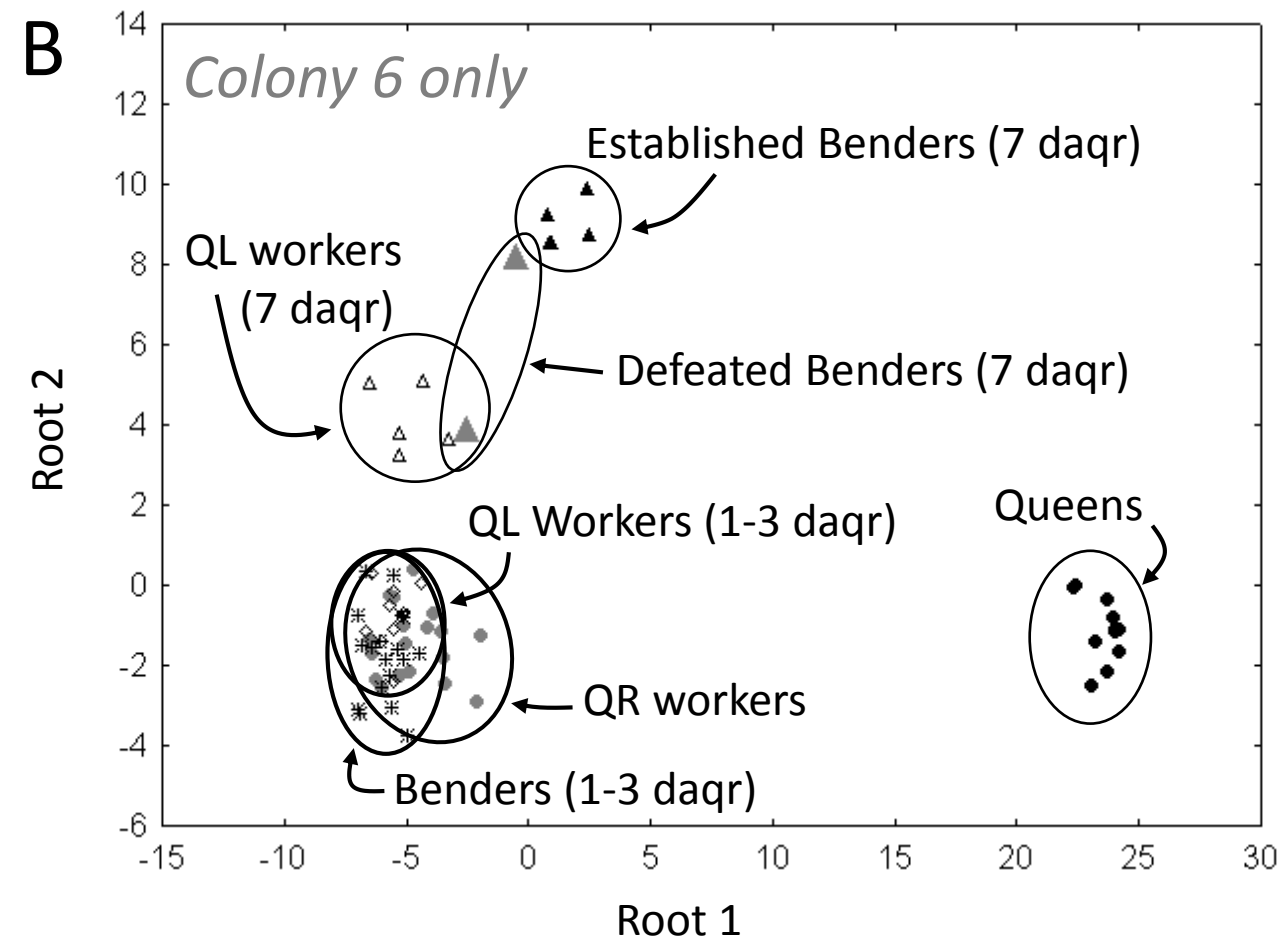

Supplement: Additional file 8: Figure S7 — Intracolonial alterations in the cuticular hydrocarbon (CHC) profile of emerging reproductives. The canonical scatterplots are based on discriminant analyses of the CHC profiles. (A) From colony 7: queens, queenright (QR) workers 4–9 days since eclosion (dse) and queenless (QL) established (Est.) benders of the same age. (B) From colony 6: queens, QR workers; QL workers and new benders 1–3 days after queen removal (daqr); and QL workers, benders and two defeated (Def.) benders 7 days after queen removal. All females from colony 6 had eclosed over a month prior. Circles encompass all profiles for a given group. [file 12983_2014_78_MOESM8_ESM.pdf]

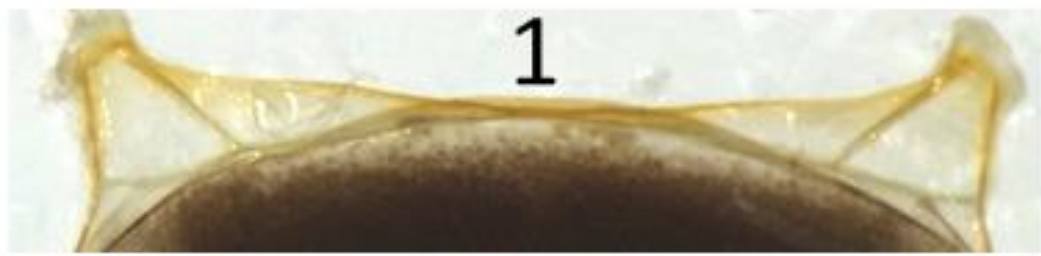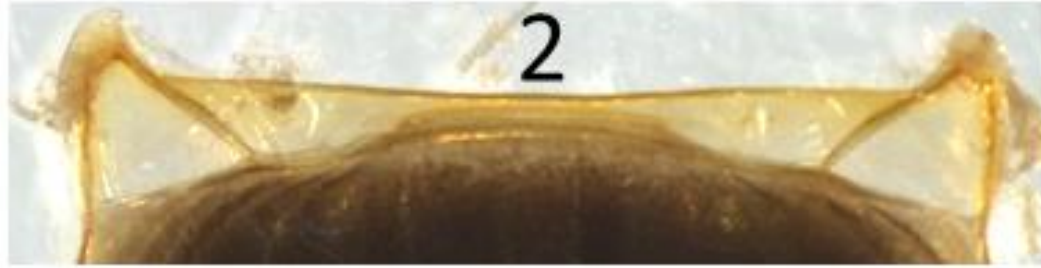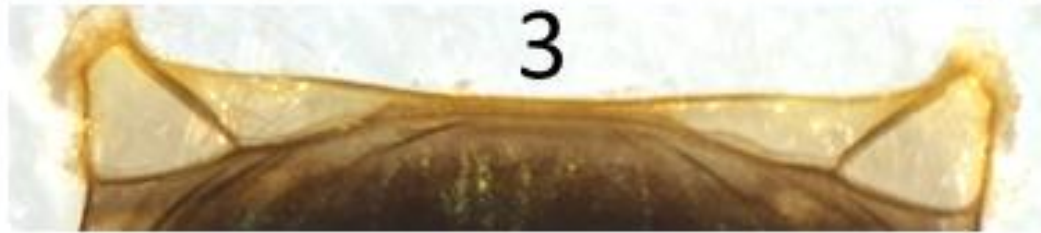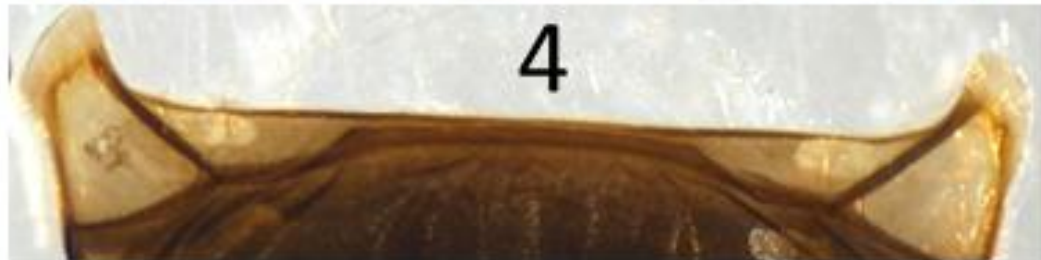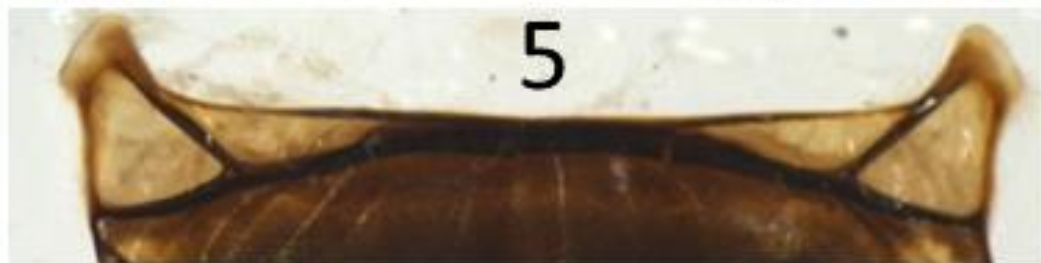

Supplement: Additional file 9: Figure S1. — Subjective measure of relative age of individuals based on the analysis of apodeme and other cuticular darkening on the anterior portion of 5th abdominal sternite. Representative samples: 1 = newly eclosed female; 2 = 7-day old builder; 3 = 12-day old worker; 4 = 20-day old forager; 5 = >35 day old forager. [file 12983_2014_78_MOESM9_ESM.pdf]
