# Supplementary material for: The role of juvenile hormone in dominance behavior, reproduction and cuticular pheromone signaling in the caste-flexible epiponine wasp, Synoeca surinama
Source: Front Zool. 2014 Oct 24;11:78. doi: 10.1186/s12983-014-0078-5 (PMC4219083; doi:10.1186/s12983-014-0078-5)
Supplement: Additional file 6: Table S1. — Mean percentage of composition and standard deviation (SD) of cuticular hydrocarbons in various types of females of Synoeca surinama. Colony (s) of origin noted on top. Ret. Time = Retention Time; dse = days since eclosion; QR = queenright; QL = queenless; D = days after queen removal. (PDF 396 kb) [file 12983_2014_78_MOESM6_ESM.pdf]

Additional Table 1

| | | | | QR All Colonies | | | | All Colonies | | QL Colony 6 | | | | | | | | QL & QR Colony 7 | | | | Colony 8 | | | |
|---|---|---|---|---|---|---|---|---|---|---|---|---|---|---|---|---|---|---|---|---|---|---|---|---|---|
| | | | | Queens | | Workers > 4 dse | | Callows (0 dse) | | Worker-turn-Benders (D1) | | Workers-stay-Workers (D1) | | Worker-turn-Benders (D7) | | Workers-stay-Workers (D7) | | Benders [QL 4-9 dse] | | Worker [QR 4-9 dse] | | New Queens | | QR Workers | |
| Peak | hydrocarbon / component | HC abrev. | Ret. Time | N=67 | | N=95 | | N=24 | | N=18 | | N=9 | | N=5 | | N=4 | | N=7 | | N=16 | | N=19 | | N=12 | |
| | | | | mean | SD | mean | SD | mean | SD | mean | SD | mean | SD | mean | SD | mean | SD | mean | SD | mean | SD | mean | SD | mean | SD |
| 1 | Heneicosane | C21 | 17.18 | 0.08 | 0.04 | 0.16 | 0.44 | 3.29 | 0.89 | 0.02 | 0.01 | 0.03 | 0.01 | 0.01 | 0.00 | 0.02 | 0.02 | 0.28 | 0.05 | 0.15 | 0.08 | 0.25 | 0.03 | 0.07 | 0.01 |
| 2 | Docosane | C22 | 19.79 | 0.06 | 0.09 | 0.33 | 0.47 | 0.61 | 0.20 | 0.05 | 0.01 | 0.05 | 0.02 | 0.03 | 0.01 | 0.02 | 0.00 | 0.12 | 0.04 | 0.10 | 0.03 | 0.08 | 0.05 | 0.11 | 0.06 |
| 3 | 9-Tricosane | C23:1 | 21.74 | 0.04 | 0.03 | 0.24 | 0.55 | 2.33 | 0.87 | 0.02 | 0.01 | 0.09 | 0.13 | 0.14 | 0.05 | 0.16 | 0.24 | 0.87 | 0.12 | 0.22 | 0.16 | 0.31 | 0.07 | 0.10 | 0.04 |
| 4 | Tricosane | C23 | 22.52 | 0.32 | 0.17 | 1.88 | 2.13 | 17.73 | 5.54 | 0.36 | 0.18 | 0.51 | 0.35 | 0.48 | 0.10 | 0.48 | 0.31 | 3.23 | 0.52 | 2.34 | 1.06 | 2.24 | 0.26 | 0.61 | 0.11 |
| 5 | Tetracosane | C24 | 25.08 | 0.19 | 0.22 | 0.72 | 1.09 | 0.63 | 0.28 | 0.27 | 0.11 | 0.25 | 0.12 | 0.25 | 0.02 | 0.15 | 0.03 | 0.27 | 0.05 | 0.34 | 0.08 | 0.27 | 0.05 | 0.18 | 0.06 |
| 6 | 9-Pentacosane | C25:1 | 27.10 | 16.92 | 5.70 | 5.95 | 5.22 | 30.57 | 7.57 | 2.19 | 0.65 | 3.37 | 3.01 | 11.36 | 2.40 | 4.85 | 3.92 | 24.84 | 1.49 | 7.99 | 3.24 | 24.11 | 1.31 | 3.34 | 0.79 |
| 7 | 11-Pentacosane | C25:2 | 27.20 | 0.22 | 0.11 | 0.28 | 0.14 | 0.73 | 0.20 | 0.24 | 0.10 | 0.25 | 0.08 | 0.38 | 0.08 | 0.34 | 0.12 | 0.53 | 0.05 | 0.30 | 0.08 | 0.49 | 0.07 | 0.28 | 0.10 |
| 8 | Pentacosane | C25 | 27.87 | 13.11 | 3.40 | 14.23 | 6.05 | 12.23 | 3.10 | 11.19 | 3.14 | 10.35 | 3.05 | 17.24 | 1.58 | 9.83 | 1.33 | 12.68 | 2.04 | 16.28 | 2.52 | 11.07 | 2.17 | 9.06 | 0.86 |
| 9 | Hexacosane | C26 | 30.10 | 0.35 | 0.26 | 1.19 | 0.76 | 0.41 | 0.33 | 0.92 | 0.33 | 0.87 | 0.41 | 0.60 | 0.10 | 0.56 | 0.10 | 0.22 | 0.05 | 0.73 | 0.08 | 0.25 | 0.05 | 0.65 | 0.13 |
| 10 | 5-Heptacosane | C27:1 | 31.96 | 2.87 | 1.04 | 4.35 | 1.54 | 4.76 | 1.11 | 4.80 | 1.41 | 4.81 | 1.53 | 7.51 | 1.73 | 6.75 | 1.98 | 5.86 | 0.58 | 4.24 | 0.54 | 4.21 | 0.43 | 3.64 | 0.85 |
| 11 | Heptacosane | C27 | 32.58 | 4.21 | 0.46 | 7.19 | 1.76 | 1.25 | 0.59 | 6.87 | 1.27 | 6.99 | 1.34 | 7.55 | 1.41 | 8.19 | 2.59 | 2.77 | 0.43 | 5.92 | 0.79 | 3.61 | 0.32 | 7.85 | 0.73 |
| 12 | Octacosane | C28 | 34.80 | 0.33 | 0.36 | 1.12 | 0.89 | 0.43 | 0.63 | 0.96 | 0.44 | 0.81 | 0.53 | 0.30 | 0.06 | 0.40 | 0.10 | 0.16 | 0.03 | 0.40 | 0.08 | 0.18 | 0.06 | 0.47 | 0.08 |
| 13 | 7-Nonacosane | C29:1 | 36.57 | 3.20 | 1.08 | 2.85 | 0.94 | 2.72 | 0.62 | 4.03 | 0.81 | 3.52 | 1.42 | 4.45 | 0.91 | 3.88 | 0.99 | 5.39 | 0.78 | 3.54 | 0.64 | 4.06 | 0.46 | 2.46 | 0.68 |
| 14 | Nonacosane | C29 | 37.17 | 9.20 | 1.43 | 7.95 | 1.68 | 1.66 | 0.88 | 8.05 | 1.53 | 7.06 | 0.98 | 8.73 | 1.89 | 8.55 | 2.71 | 4.85 | 0.59 | 7.26 | 0.77 | 6.85 | 0.61 | 7.87 | 0.60 |
| 15 | 9-Triacontane | C30:1 | 38.73 | 0.33 | 0.09 | 0.66 | 0.23 | 0.46 | 0.11 | 0.94 | 0.25 | 1.07 | 0.59 | 0.83 | 0.23 | 1.06 | 0.52 | 0.91 | 0.10 | 0.79 | 0.12 | 0.65 | 0.09 | 0.58 | 0.12 |
| 16 | Triacontane | C30 | 39.23 | 0.86 | 0.34 | 2.08 | 0.86 | 0.49 | 0.62 | 2.33 | 0.48 | 2.20 | 0.50 | 1.10 | 0.24 | 1.99 | 0.45 | 0.47 | 0.09 | 1.28 | 0.23 | 0.58 | 0.07 | 1.90 | 0.19 |
| 17 | 9-Hentriacontane | C31:1 | 41.08 | 23.97 | 2.76 | 16.55 | 3.54 | 14.14 | 3.50 | 20.54 | 3.10 | 18.90 | 3.94 | 15.68 | 3.06 | 16.32 | 3.80 | 23.66 | 2.74 | 17.76 | 1.69 | 23.46 | 1.85 | 16.84 | 2.03 |
| 18 | Hentriacontane | C31 | 41.54 | 20.71 | 6.05 | 19.03 | 5.92 | 2.24 | 1.00 | 19.69 | 3.27 | 21.20 | 6.60 | 15.95 | 3.56 | 22.12 | 6.48 | 8.55 | 1.27 | 16.45 | 2.56 | 13.85 | 1.39 | 25.37 | 2.27 |
| 19 | 9-Docotriacontane | C32:1 | 42.95 | 0.17 | 0.05 | 0.54 | 0.21 | 0.25 | 0.08 | 0.96 | 0.23 | 0.95 | 0.19 | 0.48 | 0.14 | 0.73 | 0.27 | 0.48 | 0.04 | 0.65 | 0.13 | 0.37 | 0.06 | 0.76 | 0.10 |
| 20 | Docotriacontane | C32 | 43.38 | 0.51 | 0.23 | 1.68 | 0.76 | 0.30 | 0.29 | 1.77 | 0.43 | 1.95 | 0.72 | 0.71 | 0.16 | 1.65 | 0.43 | 0.26 | 0.07 | 1.15 | 0.25 | 0.33 | 0.09 | 1.99 | 0.24 |
| 21 | 9-Tritriacontane | C33:1 | 45.18 | 0.76 | 0.27 | 5.13 | 1.85 | 2.23 | 0.82 | 8.03 | 1.66 | 7.55 | 0.58 | 3.39 | 0.79 | 5.58 | 1.09 | 2.50 | 0.20 | 6.34 | 1.05 | 1.52 | 0.24 | 6.69 | 0.94 |
| 22 | Tritriacontane | C33 | 45.67 | 1.60 | 0.50 | 5.91 | 2.18 | 0.55 | 0.27 | 5.77 | 1.31 | 7.23 | 2.68 | 2.83 | 0.74 | 6.38 | 1.64 | 1.09 | 0.36 | 5.77 | 1.15 | 1.26 | 0.22 | 9.17 | 1.15 |
