# Supplementary material for: The role of juvenile hormone in dominance behavior, reproduction and cuticular pheromone signaling in the caste-flexible epiponine wasp, Synoeca surinama
Source: Front Zool. 2014 Oct 24;11:78. doi: 10.1186/s12983-014-0078-5 (PMC4219083; doi:10.1186/s12983-014-0078-5)
Supplement: Additional file 7: Table S2. — Mean percentage of composition and standard deviation (SD) of cuticular hydrocarbons in various types of methoprene-, cyclohexane- (cyclo) non-treated females from queenright colonies 11 and 12. Ret. Time = Retention Time; dse = days since eclosion. [file 12983_2014_78_MOESM7_ESM.pdf]

Additional Table 2

				manipulation: Colony A						*manipulation: Colony B*			
				JHM-treated [7 dse]		Cyclo-treated [7 dse]		Non-treated [7 dse]		JHM-treated [7 dse]		Cyclo-treated [7 dse]	
**Peak**	**hydrocarbon / component**	**HC abrev.**	**Ret. Time**	**N=18**		**N=20**		**N=6**		**N=14**		**N=13**	
				**mean**	SD	**mean**	SD	**mean**	SD	**mean**	SD	**mean**	SD
1	Heneicosane	**C21**	17.18	**0.18**	0.08	**0.14**	0.09	**0.15**	0.11	**0.23**	0.06	**0.20**	0.09
2	Docosane	**C22**	19.79	**0.06**	0.02	**0.08**	0.08	**0.06**	0.03	**0.10**	0.02	**0.11**	0.04
3	9-Tricosane	**C23:1**	21.74	**0.62**	0.20	**0.27**	0.24	**0.30**	0.20	**0.68**	0.16	**0.41**	0.23
4	Tricosane	**C23**	22.52	**2.64**	0.67	**2.60**	1.44	**3.04**	1.62	**3.33**	0.41	**3.28**	1.16
5	Tetracosane	**C24**	25.08	**0.34**	0.07	**0.38**	0.11	**0.42**	0.13	**0.52**	0.07	**0.59**	0.13
6	9-Pentacosane	**C25:1**	27.10	**23.34**	4.50	**12.97**	6.62	**11.48**	6.33	**16.39**	1.59	**10.52**	4.48
7	11-Pentacosane	**C25:2**	27.20	**0.59**	0.10	**0.35**	0.14	**0.33**	0.14	**0.53**	0.08	**0.40**	0.17
8	Pentacosane	**C25**	27.87	**9.03**	2.68	**14.29**	3.27	**16.97**	4.68	**14.19**	4.10	**19.11**	3.88
9	Hexacosane	**C26**	30.10	**0.37**	0.14	**0.59**	0.15	**0.74**	0.14	**0.57**	0.08	**0.98**	0.23
10	5-Heptacosane	**C27:1**	31.96	**6.15**	0.90	**4.62**	0.82	**3.28**	0.41	**5.15**	0.50	**4.16**	1.03
11	Heptacosane	**C27**	32.58	**4.66**	1.23	**7.58**	1.62	**8.06**	1.90	**5.35**	0.98	**6.98**	1.61
12	Octacosane	**C28**	34.80	**0.21**	0.06	**0.37**	0.09	**0.44**	0.15	**0.29**	0.04	**0.45**	0.10
13	7-Nonacosane	**C29:1**	36.57	**4.84**	0.68	**3.13**	0.48	**2.54**	0.55	**4.04**	0.40	**2.71**	0.75
14	Nonacosane	**C29**	37.17	**7.01**	1.30	**10.17**	1.82	**11.13**	2.19	**7.65**	1.39	**8.32**	1.23
15	9-Triacontane	**C30:1**	38.73	**0.88**	0.13	**0.61**	0.13	**0.48**	0.14	**0.90**	0.17	**0.62**	0.22
16	Triacontane	**C30**	39.23	**0.74**	0.25	**1.25**	0.27	**1.42**	0.33	**1.13**	0.10	**1.66**	0.33
17	9-Hentriacontane	**C31:1**	41.08	**20.33**	2.94	**13.96**	2.41	**11.63**	2.29	**16.75**	1.95	**11.01**	3.32
18	Hentriacontane	**C31**	41.54	**11.90**	2.59	**17.89**	2.91	**18.87**	3.22	**13.11**	1.23	**16.42**	2.79
19	9-Docotriacontane	**C32:1**	42.95	**0.51**	0.07	**0.40**	0.08	**0.33**	0.08	**0.58**	0.11	**0.48**	0.18
20	Docotriacontane	**C32**	43.38	**0.47**	0.26	**0.81**	0.21	**0.92**	0.19	**0.87**	0.12	**1.43**	0.41
21	9-Tritriacontane	**C33:1**	45.18	**2.75**	0.47	**2.89**	0.90	**2.46**	0.55	**3.25**	0.85	**3.50**	1.33
22	Tritriacontane	**C33**	45.67	**2.37**	1.28	**4.66**	1.22	**4.93**	0.90	**4.38**	0.74	**6.66**	1.63
